# Comparative Characterization of Two *cxcl8* Homologs in *Oplegnathus fasciatus*: Genomic, Transcriptional and Functional Analyses

**DOI:** 10.3390/biom10101382

**Published:** 2020-09-28

**Authors:** Navaneethaiyer Umasuthan, SDNK Bathige, William Shanthakumar Thulasitha, Minyoung Oh, Jehee Lee

**Affiliations:** 1 Department of Marine Life Sciences & Fish Vaccine Research Center, Jeju National University, Jeju Self-Governing Province 63243, Korea; sanjayachem@gmail.com (S.B.); thulasitha@univ.jfn.ac.lk (W.S.T.); lucky5153my@gmail.com (M.O.); 2 Department of Ocean Sciences, Memorial University of Newfoundland, St. John’s, NL A1C 5S7, Canada; 3 Sri Lanka Institute of Nanotechnology (SLINTEC), Nanotechnology and Science Park, Mahenwatta, Pitipana, Homagama 10206, Sri Lanka; 4 Department of Zoology, Faculty of Science, University of Jaffna, Jaffna 40000, Sri Lanka; 5 Department of Biochemistry and Molecular Biology, Dalhousie University, Halifax, NS B3H 1X5, Canada; 6 Marine Science Institute, Jeju National University, Jeju Self-Governing Province 63333, Korea

**Keywords:** CXC chemokine, CXCL8, interleukin-8 (*il-8*), molecular profiling, genomic sequence arrangement, promoter prediction analyses, transcriptional expression, chemotaxis, leukocyte proliferation

## Abstract

CXCL8 (interleukin-8, IL-8) is a CXC family chemokine that recruits specific target cells and mediates inflammation and wound healing. This study reports the identification and characterization of two *cxcl8* homologs from rock bream, *Oplegnathus fasciatus*. Investigation of molecular signature, homology, phylogeny, and gene structure suggested that they belonged to lineages 1 (L1) and 3 (L3), and designated *Ofcxcl8-L1* and *Ofcxcl8-L3*. While *Ofcxcl8-L1* and *Ofcxcl8-L3* revealed quadripartite and tripartite organization, in place of the mammalian ELR (Glu-Leu-Arg) motif, their peptides harbored EMH (Glu-Met-His) and NSH (Asn-Ser-His) motifs, respectively. Transcripts of *Ofcxcl8*s were constitutively detected by Quantitative Real-Time PCR (qPCR) in 11 tissues examined, however, at different levels. *Ofcxcl8-L1* transcript robustly responded to treatments with stimulants, such as flagellin, concanavalin A, lipopolysaccharide, and poly(I:C), and pathogens, including *Edwardsiella tarda*, *Streptococcus iniae*, and rock bream iridovirus, when compared with *Ofcxcl8-L3* mRNA. The differences in the putative promoter features may partly explain the differential transcriptional modulation of *Ofcxcl8*s. Purified recombinant OfCXCL8 (rOfCXCL8) proteins were used in in vitro chemotaxis and proliferation assays. Despite the lack of ELR motif, both rOfCXCL8s exhibited leukocyte chemotactic and proliferative functions, where the potency of rOfCXCL8-L1 was robust and significant compared to that of rOfCXCL8-L3. The results, taken together, are indicative of the crucial importance of *Ofcxcl8*s in inflammatory responses and immunoregulatory roles in rock bream immunity.

## 1. Introduction

Chemokines are a superfamily of low molecular weight (8–12 kDa) chemotactic cytokine peptides, which are multifunctional chemical messengers that can activate and orchestrate the mobilization and migration of specific subsets of leukocytes along a gradient [1]. Besides their leukocyte-recruiting function toward a site of infection or injury, chemokines are now known to have pleiotropic biological effects in growth, differentiation, and immune regulation and angiogenesis, and appear to play a crucial mediator-role in linking innate and acquired arms of the immune system [2]. Functionally, chemokines are categorized into two subgroups: (1) *Homeostatic chemokines* are generally involved in lymphocyte trafficking, immune surveillance, and localization of hematopoietic precursors; (2) *Inflammatory chemokines* are only produced by cells during inflammation to prompt the migration of leukocytes towards an injured or infected site, and also to activate cells to raise an immune response and commence the wound healing process [3].

On the basis of structural properties and primary amino acid (aa) sequence, chemokines are classified into four subfamilies, including the CXC, CC, C, and CX3C subfamilies, according to the number and position of the conserved cysteine residues [4]. CC and CXC chemokines are the two major subfamilies, which are distinguished by the separation of two N-terminal cysteine residues in their primary aa sequences by a non-conserved aa (X). The CXC chemokine subfamily is further divided into two sister-groups based on whether or not the CXC-motif is preceded by a tri-peptide motif of Glu-Leu-Arg (ELR). CXC chemokines with ELR motif (ELR^+^ CXC) were determined to have a broad spectrum of roles [3], such as promoting the migration of polymorphonuclear leukocytes (PMNs, including neutrophils) and promoting angiogenesis. In contrast, CXC chemokines that lack the ELR motif (ELR^-^ CXC) are chemo-attractants for lymphocytes and monocytes and suppress angiogenesis [5,6].

CXCL8 (interleukin-8, IL-8) was the first member of the CXC chemokine family ever discovered. In mammals, a wide range of cells secrete CXCL8, and as a member of ELR^+^ CXC, it acts as a potent chemo-attractant of neutrophils, and many other immune cells, and is involved in wound healing, inflammation, oxidative (respiratory) burst, and angiogenesis [7]. CXCL8 has been shown to promote proliferation, growth, and viability of vascular endothelial cells [8] and different cancer cells (reviewed in [9]). CXCL8 exerts its biological function by binding to distinct receptors, CXCR1 and CXCR2 [10], which belong to a large family of seven transmembrane domain (TM) containing G-protein-coupled receptors (GPCRs), to transduce the signal and initiate downstream cascade. A remarkable feature of CXCL8 is its inducibility by a diverse number of stimuli, including other cytokines, bacteria, and viruses.

Several *cxcl8* homologs have been cloned from a number of species, including fish. In a phylogeny perspective, fish CXCL8 sequences can be classified under three lineages (i.e., CXCL8-L1, CXCL8-L2, and CXCL8-L3). CXCL8-L1 was considered a fish-specific CXCL8-like molecule [11] and has been identified in various species, including Japanese flounder *Paralichthys olivaceous* [12], common carp *Cyprinus carpio* [13,14,15], rainbow trout *Oncorhynchus mykiss* [16], zebra fish *Danio rerio* [17], grass carp *Ctenopharyngodon idellus* [18], ayu *Plecoglossus altivelis* [19], half-smooth tongue sole *Cynoglossus semilaevis* [20], turbot *Scophthalmus maximus* [21], and large yellow croaker *Larimichthys crocea* [22,23]. CXCL8-L2 is a cyprinid-specific lineage and has been identified in common carp [14,15], zebra fish [24], and grass carp [11,25]. Meanwhile, CXCL8-L3 is a recently identified lineage, which has been reported in large yellow croaker [26] and rainbow trout [11]. While the majority of the previous studies were primarily focused on transcriptional responses of *cxcl8* [12,17,22,27,28], some groups have also examined the biological functions of fish CXCL8, such as chemotaxis [11,13,14,15,18,19,20,21,23,26,29,30,31,32,33], cell proliferation [20,21,31], superoxide production/respiratory burst [15,22,23,26], and phagocytosis [30,32]. Carp recombinant CXCL8s, CXCa-L1 and CXCL8-L2, demonstrated chemotactic activity both in vivo and in vitro, and enhanced the respiratory burst [14,15]. In zebrafish, both Cxcl8-l1 and Cxcl8-l2 were required for normal neutrophil migratory behavior [24]. CXCL8-L3 from large yellow croaker was chemotactically active and increased the respiratory burst [26]. Besides these findings, the genomic arrangement of *cxcl8* has not been investigated in a comparative context in detail to understand the genomic evolutionary aspects of *cxcl8* [22,27,34]. Moreover, plenty of evidence in mammals indicates that flagellin could act as a potent inducer of *cxcl8* mRNA expression [35,36]. However, the impact of the flagellin-induced pathway on *cxcl8* is not completely understood in teleost fish. Aiming to understand these aspects, we characterize two *cxcl8-like* genes identified from rock bream *Oplegnathus fasciatus* (*Ofcxcl8*s), a fish species with a relatively high market value and being widely cultured in Korea, Japan, and China. Through in silico approaches, *Ofcxcl8*s are characterized at both cDNA and gDNA levels, and their genomic structures are included in the inter-species gene structural comparison. We further investigated their mRNA expression profiles in naïve fish and animals injected with immunogens (i.e., flagellin, lipopolysaccharide (LPS) and polyinosinic: polycytidylic acid [poly(I:C)]) or pathogens (bacteria and virus). The biological function of OfCXCL8 homologs was also demonstrated using the purified recombinant proteins by examining their chemotactic and proliferative activities. Collectively, this study provides some insights into molecular aspects of two *cxcl8* homologs of teleost origin.

## 2. Materials and Methods

### 2.1. Chemicals, Reagents, Experimental Fish, and Immune Stimulants

All the molecular biology grade chemicals were provided by Sigma (St. Louis, MO, USA). The kits used in different steps of cloning (i.e., polymerase chain reaction (PCR)- and gel-purification, and plasmid extraction) were obtained from Bioneer (Daejeon, Korea) or QIAGEN (Hilden, Germany). Reagents used in genomic and proteomic works, including Taq polymerase, SYBR Ex Taq, molecular markers, and restriction enzymes, were purchased from TaKaRa Bio (Shiga, Japan) or Thermo Scientific Inc. (Waltham, MA, USA). Primers were synthesized by Integrated DNA Technologies, Inc. (Coralville, IA, USA). The pMAL™ Protein Fusion and Purification System (New England Biolabs, Inc., Ipswich, MA, USA) was used in cloning and recombinant protein production. Transwell™ 24-well plates (Corning™, Corning, NY, USA) and Premix WST-1 cell-proliferation assay kit (TaKaRa) were used in functional bioassays of recombinant proteins. Rock bream fish were supplied by the Jeju Special Self-Governing Province Ocean and Fisheries Research Institute (Jeju, Korea), while ultrapure flagellin purified from *Salmonella typhimurium* (FLA-ST) was obtained from InvivoGen (San Diego, CA, USA); concanavalin A (Con A), LPS, and poly(I:C) were purchased from Sigma (St. Louis, MO, USA). Two bacterial strains (i.e., *Edwardsiella tarda* and *Streptococcus iniae*) were obtained from the Department of Aquatic Life Medicine at the Chonnam National University (Gwangju, Korea). Rock bream iridovirus (RBIV) was isolated from the kidney specimens sampled from RBIV-infected moribund rock bream.

### 2.2. Animal Rearing and Ethics

Animal rearing, immune challenge experiments, and tissue collection were performed using the research facilities in Marine Molecular Genetics Lab, Jeju National University (MMGL, JNU, Jeju, Korea), except for FLA-ST injection, which was conducted in the Fish Vaccine Center, JNU (FVC, Jeju, Korea). Fish were acclimated to the laboratory conditions (salinity 34 ± 1‰, pH 7.6 ± 0.5 at 24 ± 1 °C) for one week prior to any experiments, and fed with a commercial diet. All the pathogen/stimulant challenge experiments were conducted in accordance with The Code of Ethics of the EU Directive 2010/63/EU.

### 2.3. Determination of Complementary- and Genomic-DNA (cDNA and gDNA) Sequences of Ofcxcl8 Homologs

Homology analyses of a previously constructed multi-tissue cDNA library [37,38] by BLAST [39] identified two cDNA contigs (contig15538 and contig08530) demonstrating significant identities with known teleost *cxcl8* homologs of lineage 1 and 3, and are recognized as *Ofcxcl8-L1* and *Ofcxcl8-L3*, respectively. The primers targeting amplicons, which include the coding sequence (CDS) of each of these sequences, were used in PCR (Table S1). Each amplicon was cloned into T-Vector pMD20 (TaKaRa) and confirmed by sequencing (Macrogen, Seoul, Korea).

As we described in an earlier study [40], a bacterial artificial chromosome (BAC) library was custom-constructed for rock bream, and a PCR-based method was employed in library screening to identify the positive clones containing the putative *Ofcxcl8* sequences. The gene-specific primers were used to target the respective CDS in this two-step PCR protocol (Table S1) [40]. Two individual clones were identified and sequenced by next-generation sequencing (NGS), using the GS-FLX™ technique (Macrogen, Seoul, Korea) to determine the gDNA sequence, including 5′-proximal region of these two *Ofcxcl8* homologs.

### 2.4. Molecular Profiling of Rock Bream cxcl8 Homologs by Computational Tools

The CDS of each *Ofcxcl8* was identified and translated to obtain their putative amino acid (aa) sequence using DNAssist (2.2). The aa sequences of OfCXCL8s were examined by BLASTp suite at NCBI, and the CXCL8 homologs were retrieved to compare and compute the homology indices using the MatGAT program [41] using the BLOSUM62 matrix. Different tools available in the ExPASy Resource Portal [42] were used for domain and signature identification (e.g., ProtParam, ScanProsite, SMART, InterProScan, and Motif Scan), signal peptide prediction (SignalP), and N-glycosylation site prediction (NetNGlyc). The exon-intron distribution of *Ofcxcl8* homologs was determined based on the alignment of their corresponding gDNA and cDNA sequences using the Spidey tool [43] (now superseded by Splign). Genomic sequences of *cxcl8* from other taxonomies were obtained from GenBank [44] or Ensembl [45] databases and compared at genomic structural levels. The 5′-proximal region of each *Ofcxcl8* sequence was subjected to the JASPAR server [46], and potential transcription factor binding sites (TFBS) were located and mapped. The aa sequences of CXCL8 homologs from tetrapods and different fish species were used in molecular phylogenetic analyses. These sequences were aligned using Muscle tool, and the Neighbor-Joining (NJ) algorithm available in MEGA X platform [47] was employed in investigating the evolutionary history of CXCL8 with the support of 5000 bootstrap tests.

### 2.5. Transcriptional Profiling of Rock Bream cxcl8 Homologs

#### 2.5.1. Immune Challenge Experiments

Animals were randomly divided into eight groups, as outlined in Table S2. Rock breams with an average body weight of ~96 g were used in experiments conducted with groups 1–3 at FVC, JNU. The rest of the experiments conducted at MMGL, JNU, utilized fish with an average body weight of ~50 g. Untreated animals in the first group served as a control group and were used in the tissue mRNA profiling of *Ofcxcl8* homologs. The impact of different pathogen-associated molecular patterns (PAMPs, i.e., immune stimulants including FLA-ST, LPS, and poly(I:C)), and/or pathogens (*E. tarda* (Gram-negative), *S. iniae* (Gram-positive), and RBIV) on the transcriptional expression of *Ofcxcl8* homologs, six immune-challenges were performed. Details of challenges have been previously described (Table S2) [48,49]. The animals in the second group injected with phosphate-buffered saline (PBS; pH 7.4) served as a mock control.

#### 2.5.2. Sampling for Spatial Tissue Distribution of *Ofcxcl8* Homologs in Unchallenged Animals

The physiological distribution of *Ofcxcl8* transcripts in different tissues was determined in naïve fish. Blood was withdrawn (~1 mL/fish) from three unchallenged fish before aseptically dissecting them on ice (Table S2, Group No. 1). Specimens from different tissues, including gills, spleen, intestine, skin, head kidney, kidney, heart, liver, brain, and muscle, were then sampled. Blood samples were centrifuged immediately at 3000× *g* for 10 min at 4 °C, and peripheral blood cells (PBCs) were separated.

#### 2.5.3. Sampling for Temporal Transcriptional Analyses of *Ofcxcl8* Homologs in Immune-Challenged Animals

From FLA-ST-injected animals, whole blood was withdrawn to harvest the PBCs. Subsequently, six different tissue specimens, including head kidney, spleen, liver, intestine, gills, and kidney, were collected from four animals (*n* = 4) at 3, 6, 12, and 24 h post-injection (p.i.), from the experimental (Group no. 3) and both control (unchallenged and PBS-injected) groups (Group no. 1 and 2; Table S2). Similarly, triplicate fish (*n* = 3) were sampled and killed from the other challenge (Group no. 4–8) and control (Group no. 1 and 2) groups to excise spleen tissue specimens at 3, 6, 12, 24, and 48 h p.i. PBCs and all tissue specimens were flash-frozen in liquid nitrogen and stored at −80 °C until the RNA was extracted.

#### 2.5.4. In Vitro Concanavalin A (Con A) Stimulation of Peripheral Blood Leukocytes (PBLs)

Detailed protocols for PBL isolation and in vitro Con A stimulation have been reported in our earlier publication [10]. Briefly, the whole blood was withdrawn from rock bream and subjected to a density gradient centrifugation (OptiPrep™ (Sigma); 1.07 and 1.05 g cm^−3^) at 800× *g* for 30 min at 15 °C. PBLs at 1.07 gcm^−3^ interface were harvested and seeded onto 24-well plates (4 × 10^5^). Con A isolated from jack bean (Sigma) was used to stimulate PBLs at 70 µg/mL and incubated for 24 h at 20 °C. Untreated PBLs served as the controls. PBLs were harvested at 3, 6, 12, and 24 h post-treatment (p.t.) from both Con A-treated and untreated control wells and immediately subjected to RNA extraction using the SpinClean™ RNA Purification kit (Mbiotech, Seoul, Korea) as per the manufacturer’s instructions.

#### 2.5.5. Total RNA Extraction and cDNA Synthesis

Total RNA was extracted from each tissue specimen (~50 mg/fish) from the in vivo experiment (Section 2.5.2 and Section 2.5.3) using TRI Reagent™ (Sigma–Aldrich, USA), according to the manufacturer’s instructions. The RNA concentration and purity were spectrophotometrically determined by a UV spectrophotometer (Bio-Rad, USA). Then, 2.5 μg of purified total RNA was reverse-transcribed with the PrimeScript™ first-strand cDNA synthesis kit according to the manufacturer’s specifications (TaKaRa). Resulting cDNA was diluted 40× and stored at −20 °C until use.

#### 2.5.6. Determination of *Ofcxcl8* Transcript Levels by Quantitative Real-Time PCR (qPCR)

All the qPCR assays were performed by following the essential MIQE guidelines [50] using a Thermal Cycler Dice Real-Time System (TP800; TaKaRa). Details of primers targeting a fragment of the housekeeping gene (*β-actin*; Accession No. FJ975145) and of the target genes (*Ofcxcl8* homologs) are given in Table S1. PCRs were performed in triplicate in a final volume of 10 μL containing 3 µL of diluted cDNA, 0.4 µL of each primer (10 pmol/µL), 5 µL of 2× TaKaRa ExTaq™ SYBR premix, and 1.2 µL of PCR-grade H_2_O. The thermal profiles included one cycle of 95 °C for 30 s, followed by 40 cycles of 95 °C for 5 s, 58 °C for 10 s, and 72 °C for 20 s. Finally, dissociation curve analysis (95 °C for 15 s, 60 °C for 30 s, and 95 °C for 15 s) was performed to verify the amplification of a single product. Target specificity was also validated by examining the amplicons on 1% agarose gel. The transcription levels of *Ofcxcl8* homologs relative to that of *β-actin* were determined by the Livak method [51], as described in our earlier report [49]. The relative gene expression fold-change of *Ofcxcl8* homologs was computed with respect to the tissue that had the lowest mRNA and the control groups (i.e., PBS-injected and unchallenged) in spatial and temporal transcriptional analyses, respectively.

### 2.6. Functional Characterization of Rock Bream OfCXCL8 Recombinant Proteins

#### 2.6.1. Construction of Recombinant Plasmids

In order to express the mature OfCXCL8 recombinant proteins, partial CDSs of *Ofcxcl-8* homologs (excluding the signal peptide) were cloned into the pMAL-c2X vector using the standard PCR-based approach. Briefly, PCR was performed in a 50 μL reaction mixture containing 4 units (U) of Ex Taq polymerase (TaKaRa), 5 μL of 10× Ex Taq buffer, 4 μL of 2.5 mM dNTPs, 25 pmol of each primer appended with restriction sites for EcoRI and HindIII (Table S1), and 50 ng of cDNA (from gills for *Ofcxcl8-L1* and spleen for *Ofcxcl8-L3*) as the template. The assay was conducted with a thermal profile of initial incubation for 3 min at 94 °C; 32 cycles of 30 s denaturation at 94 °C, 30 s of annealing at 58 °C, and 30 s of elongation at 72 °C; and a final extension for 5 min at 72 °C. Purified amplicons and pMAL-c2X expression vector were individually digested with EcoRI and HindIII according to manufacturer’s instructions (TaKaRa). Each digested product was gel-purified (Accuprep™ gel purification kit, Bioneer) and ligated using T4 DNA ligase (TaKaRa), at 4 °C overnight. The in-frame insertion of each *Ofcxcl8* amplicon was confirmed by sequencing (Macrogen). Recombinant pMAL-c2X harboring fragments of *Ofcxcl8* CDSs and empty pMAL-c2X vector were individually transformed into *Escherichia coli* BL21 (DE3) cells (Novagen) for protein expression.

#### 2.6.2. Bacterial Expression of Recombinant OfCXCL8 (rOfCXCL8) Proteins

A pilot study was conducted to determine the optimal conditions for the recombinant production of rOfCXCL8 proteins, which were then over-expressed in a prokaryotic system, with maltose-binding protein (MBP) as a fusion protein, as described earlier with some modifications [52,53]. Briefly, the BL21 (DE3) cells bearing the recombinant and empty vectors were individually grown in 1 L LB medium supplemented with 100 μg/mL ampicillin and 100 mM glucose at 37 °C (200 rpm) until the optical density at 600 nm (OD_600_) was reached ~0.5. Each culture was then transferred to 17 °C, and when OD_600_ reached 0.6, isopropyl-β-thiogalactopyranoside (IPTG) was added at a final concentration of 0.3 mM. Following overnight incubation at 17 °C (200 rpm), cells were harvested by centrifugation (3500 rpm for 30 min at 4 °C), resuspended in column buffer (CB; 20 mM Tris-HCl, 200 mM NaCl, pH 7.4) and frozen at −20 °C.

#### 2.6.3. Purification and Evaluation of Recombinant OfCXCL8 (rOfCXCL8) Proteins

MBP-tagged rOfCXCL8 proteins and rMBP were individually purified by affinity chromatography as per the pMAL™ protein fusion and purification system manual. The following steps were conducted by maintaining samples on ice or at 4 °C. Briefly, bacterial pellets were thawed and lysed by cold-sonication. Cell debris and the crude extracts were separated by subjecting the lysate to centrifugation (10,000 rpm for 30 min at 4 °C). Amylose resin was mixed with the crude extracts for 30 min and gently inverted before applying onto column. The column content was washed with 12× volume of CB. Finally, rOfCXCL8-L1, rOfCXCL8-L3, and rMBP were eluted by applying elution buffer (CB + 10 mM maltose), and each protein was quantified by the Bradford method using bovine serum albumin (BSA) as the standard. The entire purification procedure was monitored by subjecting the samples collected at different steps on 12% SDS–PAGE under reducing conditions, along with molecular standards (Enzynomics), and stained with 0.05% Coomassie blue R-250.

#### 2.6.4. Cell Migration Assay

A rock bream was freshly killed, the kidney was aseptically removed, and washed with PBS. The tissue was disaggregated on a sterile metal mesh and passed through with Leibovitz’s L-15 (Sigma) medium. Cells were briefly washed by centrifuging at 1600 rpm for 5 min at 25 °C to remove tissue debris. The cell suspension was then layered on a density gradient medium (Optiprep), and centrifuged at 700× *g* for 20 min at 25 °C. Leukocytes at the interface were harvested and resuspended in L-15 (1 × 10^7^ cells/mL), and microscopically examined.

The chemotaxis assay was carried out in Transwell™ 24-well plates (Corning™). Two rOfCXCL8 proteins and rMBP were diluted with L-15 medium to 1, 10, and 100 ng/µL. A 600 µL aliquot of each dilution was pipetted into the lower chamber of Transwell apparatus. The upper chamber with a polycarbonate membrane filter (3 µm pore size) was kept on top of the lower chamber. A total of 200 µL of leukocyte suspension (2 × 10^6^ cells) was added to the upper chamber, and the apparatus was incubated at 25 °C for 3 h. The number of migrated cells in the lower chamber was counted under a microscope (Leica DMIL LED, Germany) using a hemocytometer. The assay was repeated three times independently. The chemotactic activity of each protein is presented as ‘chemotactic index (ci)’ (ci = number of cells migrated in response to rOfCXCL8 or rMBP/ number of cells migrated in the presence of elution buffer (negative control)).

#### 2.6.5. Cell Proliferation Assay

A 100 µL leukocyte suspension was pipetted into a 96-well plate (1 × 10^6^ cells/well). An equal volume of rOfCXCL8 proteins and rMBP diluted in L-15 medium to 1, 10, and 100 ng/µL or elution buffer was added to each well, and the plate was incubated at 25 °C. After two days, 100 µL of Premix WST-1 was added to each well and incubated at 25 °C for 2 h. Finally, the OD_450_ was measured, with a reference wavelength of 690 nm, using a microplate reader (Mutiskan GO, Thermo Scientific, USA). The degree of cell proliferation was presented in terms of OD_450_. The assay was performed in triplicate for each treatment.

### 2.7. Statistical Analysis

All the data were presented as mean value ± standard deviation (SD) for at least triplicate assays. Data were also subjected to one-way analysis of variance (ANOVA), and mean comparisons were conducted by Tukey’s post hoc test using IBM^®^ SPSS^®^ Statistics 25.0. The *p*-values less than 0.05 were considered statistically significant.

## 3. Results

### 3.1. Molecular Profiling of Structural Features

#### 3.1.1. Identification of Rock Bream cDNAs for *cxcl8* Homologs and Their Features

In addition to two full-length cDNA of *il-8* (*cxcl8*)-like sequences available in GenBank (accession number: AB703273 [54] and ADK35757), we retrieved two more sequences (identifiers: contig15538 and contig08530) from a transcriptomic database of rock bream, demonstrating significant homology with known *cxcl8*s, and confirmed them by PCR verification. Based on BLAST and comparative analyses, these two sequences were identified as belonging to lineages 1 (L1) and 3 (L3), and hence designated as *Ofcxcl8-L1* and *Ofcxcl8-L3*, respectively. The nucleotide information of these two homologs has been deposited in GenBank under the accession numbers of KC522966 and KC522965, respectively. The cDNA nucleotide (nt) and deduced amino acid (aa) sequences of these two rock bream interleukin-8 homologs are presented in Figure S1.

**Figure 1:**
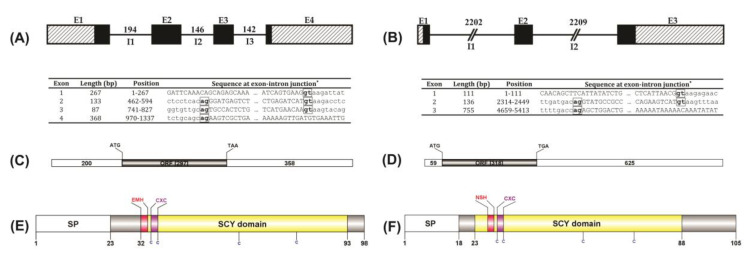
Structures of genomic organization, transcript, and protein domain architecture for rock bream interleukin-8 (*Ofcxcl8*) homologs. (**A**,**B**) Genomic composition of *Ofcxcl8*s: Exons (E1-E3/E4) and untranslated sequences are indicated by black and cross-checked boxes, respectively, whereas introns are shown as black lines with the corresponding sizes (bp). Inset table shows the exon−intron composition and features of exon−intron boundaries. *, Intron sequences are shown in lower case. The acceptor and donor sites in intron sequences are bold-boxed. (**C**,**D**) Structure of the transcripts of *Ofcxcl8* homologs: Size of coding sequences (CDS) and untranslated regions (UTRs) are shown. (**E**,**F**) The schematic diagram of rock bream CXCL8 proteins: Signal peptide (SP) is indicated by a blank box. SCY domain (CXC chemokine module; yellow), ELR motif variants (NSH or EMH; red), and CXC signature (CXC; purple) are color shaded. Four cysteine residues are marked. All motifs are shown in colors corresponding to Figure S1.

The full-length cDNAs of *Ofcxcl8-L1* (855 bp) and *Ofcxcl8-L3* (1002 bp) possessed coding sequences of 297 bp and 318 bp, flanked by 5’-untranslated regions (UTRs) of 200 bp and 59 bp, and 3’-UTRs of 358 bp and 625 bp, respectively. There were three and two mRNA instability motifs present in *Ofcxcl8-L1* and *Ofcxcl8-L3*, respectively. Both of them had a consensus polyadenylation signal (AATAAA) (Figure 1 and Figure S1).

#### 3.1.2. Recovery of Rock Bream *cxcl8* Genomic Sequences and Their Genomic Structures

A two-step PCR-based screening approach was adopted to screen a BAC genomic library with gene-specific primers, and two positive clones containing the above two *cxcl8* homologs were identified and sequenced. Genomic sequences of *Ofcxcl8-L1* and *Ofcxcl8-L3* were 1337 bp and 5413 bp, respectively. Two *Ofcxcl8* homologs identified in the current study revealed distinct genomic organizations. *Ofcxcl8-L1* and *Ofcxcl8-L3* had quadripartite (4 exons−3 introns) and tripartite (3 exons−2 introns) gene structures, and their CDSs were distributed within four and three exons, respectively (Figure 1A,B). All the intron−exon splice junctions were consistent with the GT/AG rule (Figure 1; Inset tables). Each intron of *Ofcxcl8-L3* was >2.2 kb. In contrast, *Ofcxcl8-L1* had relatively shorter introns of <200 bp; and hence the total span of the *Ofcxcl8-L1* gene was only ~1.4 kb, indicating that *Ofcxcl8-L1* gene is more compact than *Ofcxcl8-L3* (Figure 1).

#### 3.1.3. Amino Acid Sequences of Rock Bream CXCL8 Proteins: Homology and Phylogeny

The CDSs of *Ofcxcl8-L1* and *Ofcxcl8-L3* were translated into an aa sequence of 98 residues (10.8 kDa, pI 8.78) and 105 residues (11.5 kDa, pI 8.95), respectively (Figure 1E,F). Both CXCL8 homologs had N-terminal signal peptides of 23 and 28 residues, respectively. In addition, the SMART server located an SCY domain (CXC chemokine module) in OfCXCL8-L1 (32–93 aa) and OfCXCL8-L3 (23–88 aa). They harbored four invariant Cys residues, among which the first two formed the classic CXC signature motif. The typical mammalian ELR motif was replaced with the ^32^EMH^34^ motif in OfCXCL8-L1 and ^27^NSH^29^ motif in OfCXCL8-L3.

**Figure 2:**
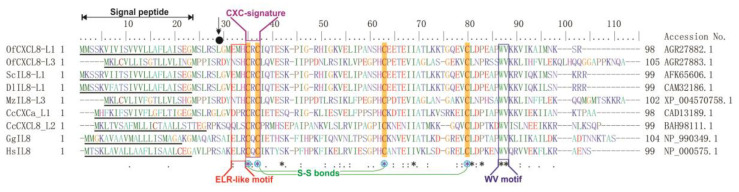
Multiple amino acid alignment analysis of rock bream interleukin-8 homologs (OfCXCL8s) with other known teleost, chicken, and human CXCL8s generated by ClustalW with default parameters. Completely (100%) and strongly conserved residues are marked with an asterisk (*) and colon (:), respectively. Weak conservation is marked by a full stop (.). The N-terminal signal peptide is underlined. Conserved Cys residues are highlighted and marked with blue asterisks, and the disulfide bonds are shown. ELR-like motif and CXC signature are boxed and indicated. A Leu residue, which is proposed to be essential for the chemotactic activity [55], is shown with a downward arrow.

Multiple sequence alignment (MSA) of CXCL8 sequences from different selected tetrapod classes disclosed their conserved and lineage-specific characteristics (Figure 2). All the CXCL8s had a signal peptide and four conserved Cys residues, two of which were separated by a non-conserved aa and formed the typical CXC signature. Fish CXCL8s lacked the ELR motif and contained an incomplete ELR motif, in which certain aa(s) had been substituted. A WV motif was conserved across all the species used in MSA. The sixth Leu residue in mature teleost CXCL8 was conserved in OfCXCL8-L1; but, not in OfCXCL8-L3, in which it was replaced with Arg (Figure 2).

The two OfCXCL8 homologs were quite different from each other in terms of aa sequence with only ~30% identity. Pairwise sequence comparison of two OfCXCL8s revealed that they share relatively higher homology with the teleost CXCL8 homologs (Table 1). The highest identities were shared by OfCXCL8-L1 and OfCXCL8-L3 with CXCL8 homologs of *Siniperca chuatsi* (88.9%) and *Lateolabrax japonicus* (91.4%), respectively. A previously deposited rock bream IL-8 sequence varied by a single aa (98% identical) from OfCXCL8-L1 [54]. Human homolog was 57.6% and 56.2% similar to OfCXCL8-L1 and OfCXCL8-L3, respectively. We also compared the aa sequences of OfCXCL8 homologs with L1 and L2 of cyprinids, where the CXCL8-L2 is a cyprinid-specific lineage [14,15]. The overall similarity between OfCXCL8-L1 and L1 members (~70%) was much higher than that of L2 (~45%). Although OfCXCL8-L3 also presented a similar trend, the overall similarity presented by OfCXCL8-L3 with both L1 and L2 was quite similar and low (i.e., ~42–55%) (Table S3). These data suggest that OfCXCL8-L1 belongs to lineage 1, whereas OfCXCL8-L3 is distinct from members of L1 and L2 lineages.

**Figure 3:**
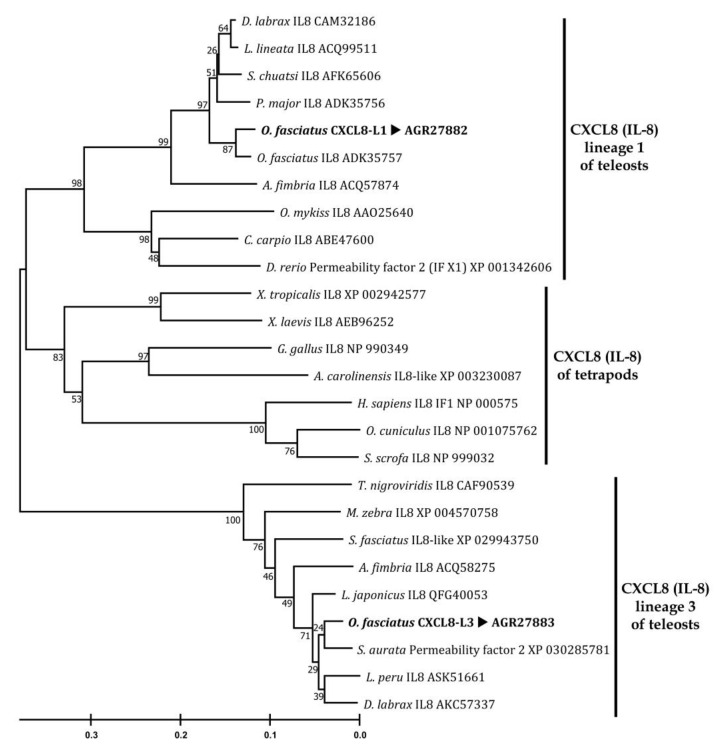
Molecular phylogenetic tree of selected CXCL8 (IL-8) homologs from vertebrates. The evolutionary history was inferred by the Neighbor-Joining method, and the evolutionary distances were computed with the p-distance method using MEGA X. Major clusters are indicated with vertical bars. The values at the forks indicate the percentage of trees in which the grouping occurred after bootstrap 5000 replicates. The tree was drawn to scale. The GenBank accession numbers are given next to each species.

In order to evaluate the evolutionary relationship of OfCXCL8s with other IL-8s, a phylogenetic tree was constructed using the Neighbor-Joining algorithm (Figure 3). There were three major clusters. Tetrapod ELR+ IL-8 orthologs and a group of teleost IL-8 orthologs formed two main clusters. OfCXCL8-L1 and the previously identified rock bream IL-8 [54] were placed within the teleost sub-cluster and shared their position with other IL-8 sequences (with significant bootstrap support (97%)) that demonstrated higher homology (Table 1). This sub-cluster represented the CXCL8-L1 homologs from teleosts. It was intriguing to note that another third cluster, diverging from these two branches with a significant evolutionary distance, was the root of the phylogenetic tree (Figure 3). This third cluster consisted of OfCXCL8-L3 and other IL-8-like sequences those shared notable homology with OfCXCL8-L3 (Table 1), and represented the teleost CXCL8-L3.

### 3.2. Profiling of Genomic Arrangement and Promoter Sequences

#### 3.2.1. Genomic Comparison of Vertebrate Orthologs of *cxcl8* (*il-8*) Gene

**Figure 4:**
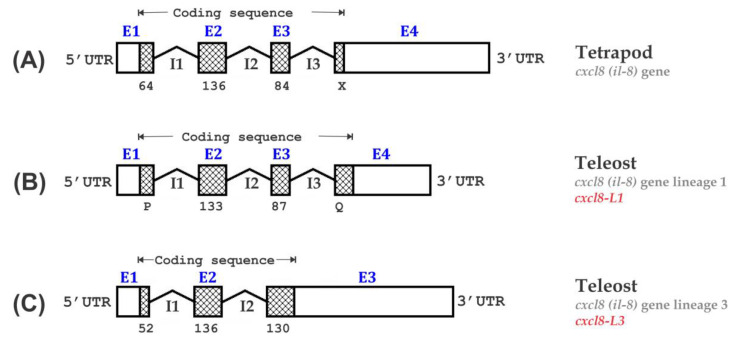
Schematic representation and comparison of the genomic organization of *cxcl8 (il-8)* in vertebrates: (**A**) tetrapods and (**B**,**C**) teleosts. While the coding sequences (CDSs) are represented by cross-checked boxes, untranslated regions (UTRs) are indicated with plain boxes. Introns are presented as bent lines with intron numbers below. Sizes of exons are indicated below the boxes if they are conserved, and otherwise, a letter is placed (P, Q, X). For more information, including the accession numbers and size of exons/introns of each sequence, please refer to Table 2.

We surveyed the exon-intron organization of *cxcl8* and compared the genomic arrangement of *Ofcxcl8* homologs with their *il-8* counterparts of different selected vertebrate origins (Figure 4 and Table 2). Genomic structurally, tetrapod *cxcl8* genes and *cxcl8-L1* genes of teleosts were similar and composed of four exons and three introns (Figure 4A,B). In general, tetrapod *cxcl8* orthologs had three conserved exons with the size of 64, 136, 84 bps, and the last exon that varied in size. Gene structure of teleost *cxcl8-L1* orthologs had two conserved middle exons of 133 and 87 bps, whereas the size of the terminal exons varied. The *Ofcxcl8-L1* exhibited this typical structure of the gene arrangement of *cxcl8-L1* (Figure 4B and Table 2). In contrast, three *cxcl8* sequences of fish origin (i.e., rock bream, tetraodon, and zebra mbuna) demonstrated a putative gene structure, which has not been reported in any species so far (Figure 4C). This novel structure was characterized by three exons of 52, 136, and 130 bps (Table 2). This comparison supported our hypothesis based on the phylogenetic analysis, and confirmed that these sequences represent the teleost *cxcl8-L3* by revealing the distinct gene structure of L3.

#### 3.2.2. Putative Promoter Sequences of Rock Bream *cxcl8* (*il-8*) Homologs

**Figure 5:**
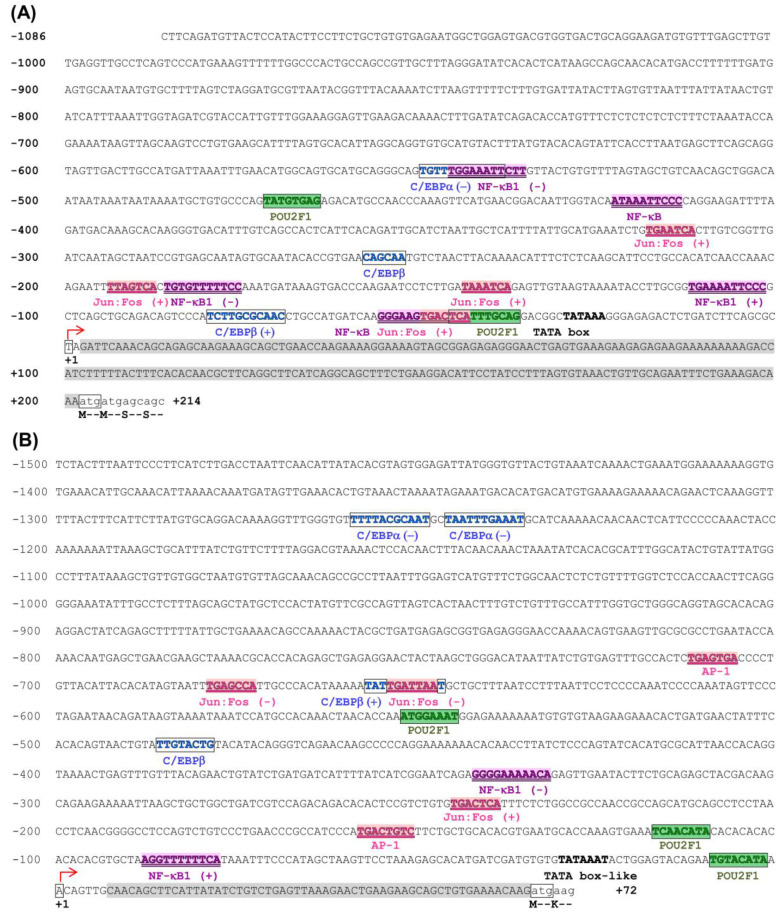
Analysis of the putative promoter region of rock bream *Ofcxcl8*s: (**A**) *Ofcxcl8-L1* and (**B**) *Ofcxcl8-L3*. The sequence numbers are relative to the predicted transcription-initiation site (TIS; +1), which is indicated by a bent-arrow. TATA box (or like) sequences preceding the transcription initiation site (TIS) are marked. Possible NF-κB/ NF-κB1, C/EBPα, C/EBPβ, Jun:Fos (AP1), and POU2F1 (Oct-1) binding sites are differentially marked and indicated below, with the directions whenever possible. The transcribed sequence up to translation start site (atg) is shown with gray shade (5′ UTR). Translation start site is boxed, and few translated aa are shown.

In order to understand the features related to transcriptional regulation, we recovered and analyzed the 5’-flanking regions of the *Ofcxcl8* homologs from the genomic sequencing (Figure 5). Inspection of these putative promoter sequences revealed the presence of a canonical TATA box sequence (TATAAA) in *Ofcxcl8-L1* and a putative TATA box-like element (TATAAAT) in *Ofcxcl8-L3*, ~25 bp upstream of transcription initiation site (TIS; +1). These sequences were also subjected to the prediction of TFBS. Results revealed the presence of several important TFBS, including NF-κB/ NF-κB1, C/EBPα, C/EBPβ (NF-IL6), Jun:Fos (AP1), and POU2F1 (Oct-1) in 5′-flanking regions of both *Ofcxcl8*s and suggested that they are under tight regulatory control mediated by multiple TFs.

### 3.3. Profiling of Transcriptional Gene Expression of Rock Bream cxcl8 (il-8) Homologs

#### 3.3.1. Tissue mRNA Expression of *Ofcxcl8* Homologs in Unchallenged Animals

**Figure 6:**
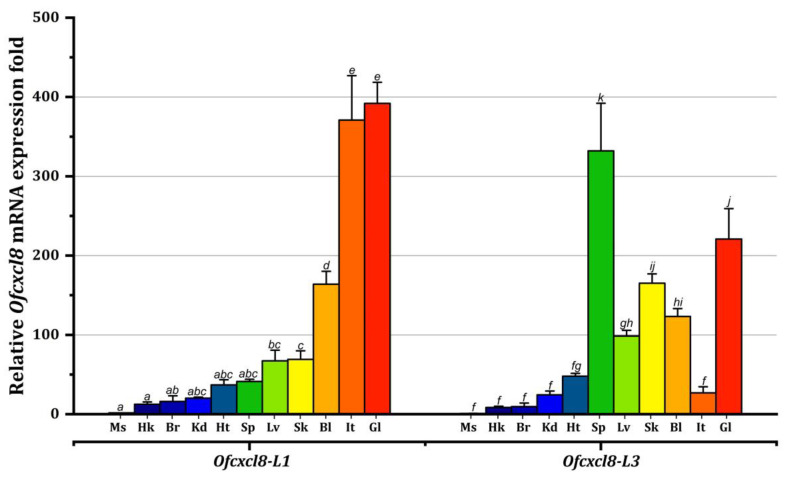
Constitutive mRNA expression of rock bream *cxcl8* (*Ofcxcl8*) homologs, *Ofcxcl8-L1* and *Ofcxcl8-L3*, in tissues of healthy juveniles. Relative transcript level of *Ofcxcl8*s was examined in 11 tissues by SYBR green qPCR. Rock bream *β-actin* was chosen as the internal reference gene. The calculation was performed using the Livak method [51], and values were calibrated against mRNA level of *Ofcxcl8-L3* in muscle. The results are reported as mean ± standard deviation (SD) of triplicates. Ms, muscle; Hk, head kidney; Br, brain; Kd, kidney; Ht, heart; Sp, spleen; Lv, liver; Sk, skin; Bl, blood (PBCs); It, intestine; Gl, gill. Different letters above bars (*Ofcxcl8-L1,* a-e and *Ofcxcl8-L3,* f-j) represent significant differences between tissue mRNA levels (one-way analysis of variance (ANOVA), Tukey’s post hoc test, *p* < 0.05).

The constitutive tissue mRNA expression of *Ofcxcl8-L1* and *Ofcxcl8-L3* was examined by qPCR using tissues collected from normal juveniles. Further analyses showed that both *Ofcxcl8* homologs were constitutively transcribed at detectable, albeit different levels in 11 tissues (Figure 6). Ubiquitous transcription of *Ofcxcl8-L1* was significantly (*p* < 0.05) higher in gills, followed by intestines and PBCs. It was transcribed at comparatively lower levels in other tissues (Figure 6, left). In contrast, significantly higher abundance of *Ofcxcl8-L3* transcripts was detected in spleen and gills (*p* < 0.05) (Figure 6, right). Meanwhile, moderate quantities of *Ofcxcl8-L3* mRNA were present in skin, blood, and liver. The other examined tissues poorly transcribed *Ofcxcl8-L3*. Our overall results revealed that the tissue mRNA profiles of both *Ofcxcl8*s are contrasting and distinct from each other (Figure 6).

#### 3.3.2. Detection of *Ofcxcl8* mRNAs in Fish Injected with FLA-ST

**Figure 7:**
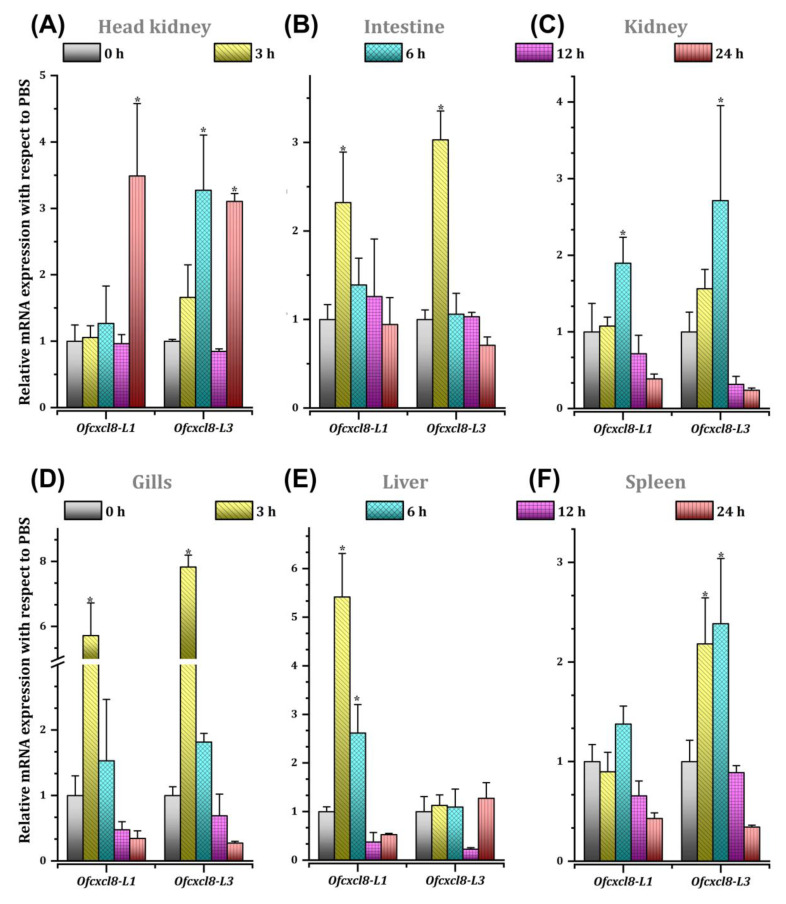
The temporal mRNA expression of rock bream *cxcl8* (*Ofcxcl8*) homologs, *Ofcxcl8-L1* and *Ofcxcl8-L3*, after flagellin (FLA-ST) injection detected by SYBR green qPCR in different tissues: (**A**) head kidney, (**B**) intestine, (**C**), kidney, (**D**) gills, (**E**) liver and (**F**) spleen. The relative mRNA level of both transcripts in each tissue was calculated by the comparative Ct method using *β-actin* as the reference gene. The fold-change in mRNA expression is presented as relative to mRNA level of PBS-injected group at each time point. Relative expression level in unchallenged (0 h) control was considered as the baseline (1). The vertical bars represent SD (*n* = 4). Statistical analysis was performed by one-way ANOVA, followed by Tukey’s post hoc test. The asterisk symbol (*) indicates the statistical difference in transcription when compared with untreated (0 h) control at *p* < 0.05.

We examined the effect of FLA-ST administration on mRNA levels of *Ofcxcl8* homologs in different rock bream tissues. A differential inductive pattern was noticed for *Ofcxcl8-L1* and *Ofcxcl8-L3* in six tissues, as detected by the qPCR (Figure 7). The mRNA levels of both *Ofcxcl8* homologs significantly increased in head kidney (24 h p.i.; and 6 h p.i. for *Ofcxcl8-L3* only), intestine (3 h p.i.), kidney (6 h p.i.), and gills (3 h p.i.) (*p* < 0.05; Figure 7A–D). Tissue-specific transcriptional modulation of *Ofcxcl8* homologs was observed in the liver (up-regulation of *Ofcxcl8-L1* at 3 h and 6 h p.i.) and spleen (up-regulation of *Ofcxcl8-L3* at 3 h and 6 h p.i.) (*p* < 0.05; Figure 7E,F).

#### 3.3.3. Detection of *Ofcxcl8* mRNAs in Fish Injected with LPS, Poly(I:C), and Pathogens

**Figure 8:**
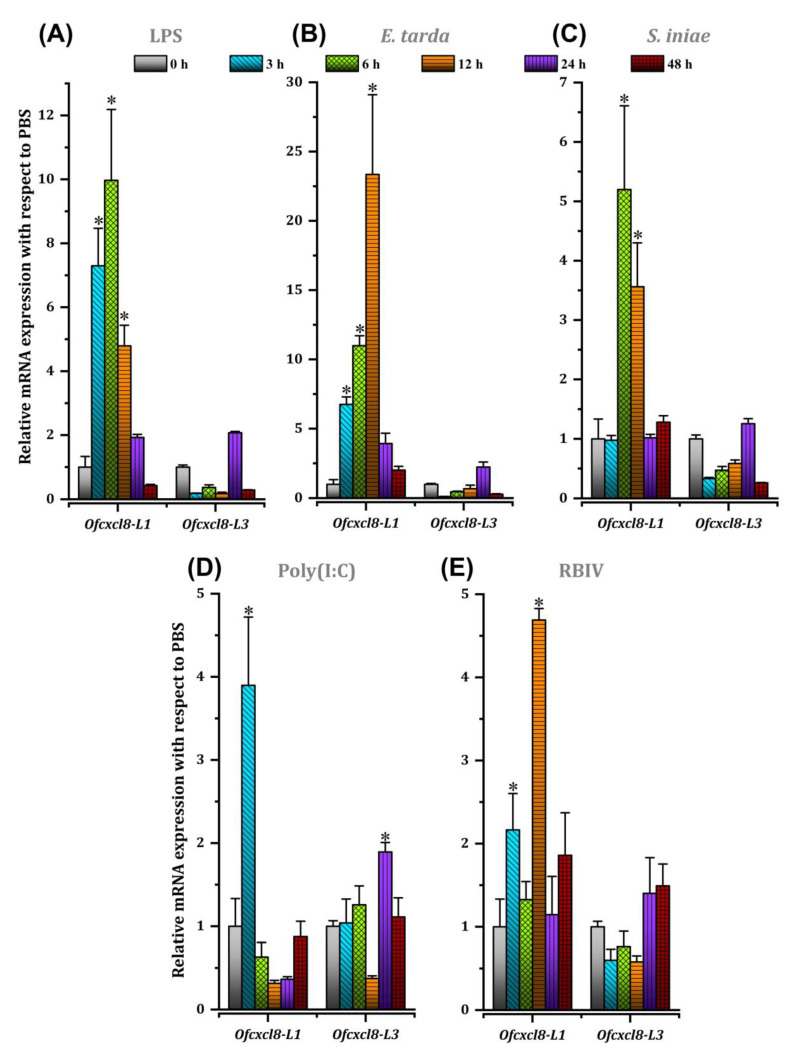
The temporal mRNA expression of rock bream *cxcl8-L1* (*Ofcxcl8-L1*) and *cxcl8-L3* (*Ofcxcl8-L3*) in spleen following immune challenges detected by SYBR green qPCR. The data refer to (**A**) LPS, (**B**) *E. tarda*, (**C**) *S. iniae*, (**D**) poly(I:C) and (**E**) RBIV. The vertical bars represent SD (*n* = 3). For details of the captions, please refer to Figure 7. The asterisk symbol (*) indicates the statistical difference in transcription when compared with untreated (0 h) control at *p* < 0.05.

The mRNA expression of *Ofcxcl8* homologs was also investigated in the spleen tissue of animals challenged with two other immune stimulants, representing Gram-negative bacterial (LPS)- and double-stranded RNA virus [poly(I:C)]-infections, and three potent pathogens (*E. tarda, S. iniae*, and RBIV) that devastate the rock bream farming. Transcriptional profiles of *Ofcxcl8-L1* and *Ofcxcl8-L3* in challenged fish are shown in Figure 8. The magnitude of *Ofcxcl8-L1* induction was higher than that of *Ofcxcl8-L3* against all the examined challenge conditions. The *Ofcxcl8-L1* mRNA was significantly (*p* < 0.05) and robustly upregulated by LPS (7.3-,10- and 4.8-fold at 3, 6, 12 h p.i.), *E. tarda* (6.8-, 11- and 23.3-fold at 3, 6, 12 h p.i.) and *S. iniae* (5.2 and 3.6-fold at 6 and 12 h p.i.) compared to unchallenged control (Figure 8A–C). However, the maximum induction of *Ofcxcl8-L1* by poly(I:C) (3.9-fold; 3 h p.i.) and RBIV (4.7-fold; 12 h p.i.) was only < 5-fold compared to unchallenged control (*p* < 0.05; Figure 8D,E). In contrast to the clear up-regulated transcriptional response of *Ofcxcl8-L1*, its counterpart *Ofcxcl8-L3* demonstrated no significant changes except for against poly(I:C) challenge (1.9-fold; 24 h p.i.) (*p* < 0.05; Figure 8D).

#### 3.3.4. Detection of *Ofcxcl8* mRNAs in Con A-stimulated PBLs

We isolated fresh PBLs and stimulated them with Con A to examine the transcriptional kinetics of *Ofcxcl8* mRNAs at different time points (Figure S2). Significantly up-regulated transcript level was detected only for *Ofcxcl8-L1* at 6-24 h p.t, and the highest level of transcription was noticed at 12 h p.t. (~2.5-fold; *p* < 0.05). Transcription of *Ofcxcl8-L3* was down-regulated across sampling time points, although it was only significant at 3, 12, and 24 h p.t. (*p* < 0.05).

### 3.4. Functional Characterization of Rock Bream CXCL8 Homologs Using Recombinant Proteins

#### 3.4.1. Bacterial Expression of Recombinant Proteins and Purification

The sequences encoding mature OfCXCL8 proteins were individually cloned into a pMAL-c2X vector that possesses a sequence encoding a fusion protein (MBP) under a strong promoter (*tac*). The *E. coli* BL21 (DE3) system was used to in vitro express the MBP-tagged rOfCXCL8 proteins through IPTG-driven induction, and rOfCXCL8 proteins were purified to homogeneity by amylose affinity chromatography. Sample fractions collected during the recombinant expression and purification of rOfCXCL8 were examined by SDS-PAGE under reducing conditions (Figure S3). A distinct and prominent band was observed in IPTG-induced cellular lysate compared with uninduced cellular extract (Figure S3; lanes L and U). The soluble fraction (Lane S) was then subjected to chromatography to purify the MBP-tagged rOfCXCL8s (Lanes F1–F5). Both rOfCXCL8 fusion proteins demonstrated a molecular mass of ~52 kDa (Figure S3), which was in agreement with the predicted masses of mature peptides (i.e., rOfCXCL8-L1, 8.8 kDa; rOfCXCL8-L3, 9.7 kDa; MBP-tag, 42.5 kDa). The MBP fusion tag was also separately purified and used as a control in biological assays.

#### 3.4.2. Chemotactic Response of Leukocytes Towards Recombinant OfCXCL8 Proteins

**Figure 9:**
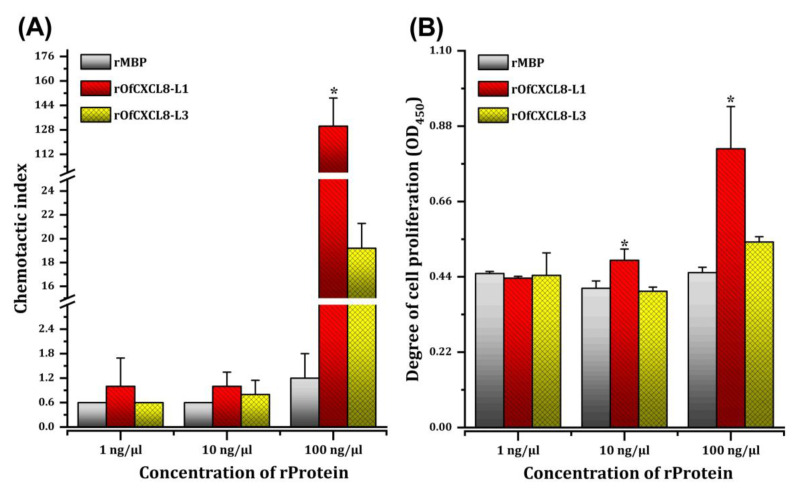
Functional characterization of recombinant OfCXCL8-L1 (rOfCXCL8-L1) and OfCXCL8-L3 (rOfCXCL8-L3) using biological assays. Kidney leukocytes were treated with three different concentrations of rOfCXCL8 proteins. The rMBP was used as a control in each assay with the same concentrations. (**A**) Chemotaxis assay was performed with Transwell to quantify the migrating leukocytes. Chemotactic index (ci) was estimated as the ratio between the number of cells migrated in response to rOfCXCL8 or rMBP and elution buffer (negative control). (**B**) Proliferation assay was performed by treating leukocytes with different concentrations of rOfCXCL8 or rMBP, and using WST-1 assay. The OD_450_ represents the degree of leukocyte proliferation. Data are presented as means ± SD (*n* = 3). Statistical analysis was performed by one-way ANOVA, followed by Tukey’s post hoc test. Significance difference between treatments with rOfCXCL8-L1 or rOfCXCL8-L3 and rMBP within a particular concentration of recombinant protein (rProtein) is indicated by an asterisk symbol (*) at *p* < 0.05.

The rOfCXCL8 proteins were tested to see if they were capable of recruiting leukocyte-enriched kidney cells in a chemotaxis assay using a Transwell apparatus. We examined the leukocyte migration induced by rOfCXCL8 proteins at three different concentrations of 1, 10, and 100 ng/µL. Our results showed that both rOfCXCL8-L1 and rOfCXCL8-L3, but not the rMBP tag, induced the leukocyte migration, where the chemotactic index of rOfCXCL8-L1 was always higher than that of rOfCXCL8-L3 (Figure 9A). The chemotactic index of rOfCXCL8-L1 was similar at 1 ng/µL and 10 ng/µL, but significantly (*p* < 0.05) higher than that of both rMBP and rOfCXCL8-L3 at 100 ng/µL. The rOfCXCL8-L3 demonstrated a dose-dependent chemotactic activity, which was not significantly different compared with rMBP control (*p* > 0.05; Figure 9A).

#### 3.4.3. Induction of Leukocyte Proliferation by Recombinant OfCXCL8 Proteins

We also examined if the rOfCXCL8 proteins had any impact on the proliferation of kidney leukocytes using the WST-1 assay. Results indicated that rOfCXCL8-L1 stimulated the leukocyte proliferation in a dose-dependent manner, where it demonstrated significant (*p* < 0.05) increase in proliferation at 10 ng/µL and 100 ng/µL compared with respective concentrations of rMBP (Figure 9B). Although rOfCXCL8-L3 exhibited slight increase in OD_450_ at 100 ng/µL (*p* > 0.05), no significant difference in proliferation between leukocytes treated with rOfCXCL8-L3 and rMBP was evident (Figure 9B).

## 4. Discussion

Chemokines are secretory proteins with pleiotropic functions associated with growth, differentiation, and activations that coordinate the nature of immune responses. Numerous genes encoding chemokines and their cognate receptors from mammals have been discovered and characterized at the molecular level. These efforts helped to extensively study the mammalian chemokine system to understand its functional architecture. Since primitive vertebrates, including fish, lack a sophisticated adaptive immune system and solely depend on the innate immune system, the roles played by chemokines in the innate immune system are inevitable [56]. Teleost orthologs of chemokine and their receptor genes remain mostly unidentified. However, emerging genomic research in fish models bolsters rapid progress in the discovery of fish chemokine networks [57].

CXCL8 (IL-8), which is the first and one of the most intensively researched chemokines, represents the prototypical chemokine of the CXC subfamily [58]. The first reported non-mammalian CXCL8 chemokine is from European river lamprey, *Lampetra fluviatilis* [59], following which many teleost *cxcl8* homologs have been identified from different species [12,13,16,27,29]. It is an angiogenic chemokine and binds with both CXCR1 and CXCR2 receptors with high affinity. We have previously identified and reported the molecular properties of these CXCL8-receptors from *O. fasciatus* [10]. With the objective of exploring the immune signaling mechanisms in fish, we have also previously characterized several immune genes associated with toll-like receptor (TLR) signaling in *O. fasciatus* [10,48,49,60,61,62,63]. In this study, two *cxcl8* homologs, *Ofcxcl8-L1* and *OfCXCL8-L3*, were isolated from *O. fasciatus*, in addition to the sequences available in GenBank (i.e., AB703273 and ADK35757), and cloned. We categorized the two *Ofcxcl8* homologs identified in this study under the lineages 1 (L1) and 3 (L3) based on their peculiar molecular features, such as signature motif (ELR-like domain), sequence homology, phylogenic clustering, and exon-intron architecture. Subsequently, two homologs identified in the current study were characterized in terms of (a) exon-intron organization, in a comparative context, with respect to vertebrate *cxcl8*s, (b) features of aa sequences, (c) characteristics of 5′-flanking regions, (d) basal tissue mRNA expression, (e) response upon in vitro con A treatment, (f) temporal-expression upon injection of PAMP and/or pathogen, and (g) biological activities using recombinant proteins.

The cDNA and aa sequences of these two *Ofcxcl8* orthologs featured several properties of known *cxcl8* members (Table 3). AU-rich elements mediating the mRNA degradation are a common characteristic of cytokines [64,65], since these molecules exist transiently and degrade immediately. The presence of multiple mRNA instability motifs (ATTTA), as found in other fish [16,27], suggest that *Ofcxcl8* transcripts may undergo rapid turnover. Putative translatable CDSs of *Ofcxcl8-L1* and *Ofcxcl8-L3* represented the information for 98 aa and 105 aa, respectively. OfCXCL8s were predicted to have an N-terminal signal peptide indicating that they are extracellularly secreted proteins. Moreover, the SCY/CXC chemokine module spanned almost the entire mature peptides of OfCXCL8s.

The ELR motif in mammalian CXC chemokines was determined to be critical for receptor binding, chemotactic activity, and angiogenesis [66,67,68]. Located in the N-terminus, the ELR motif induces signals upon binding with its receptor. For instance, when the ELR motif of mammalian CXCL8 was substituted with DLR motif, a ~100-fold reduction was noticed in biological activity, indicating that, although the DLR motif is functional, motif-replacement markedly reduced the chemotactic potential of CXCL8 [69]. As a consequence of its importance, all the tetrapod CXCL8 orthologs possess this characteristic ELR motif. In contrast to tetrapod CXCL8s, as found in many other reported teleostean CXCL8s [70], OfCXCL8s possessed ELR motif variants, EMH and NSH tripeptide sequences in OfCXCL8-L1 and OfCXCL8-L3, respectively. Among all the teleost CXCL8s characterized, homologs with the ELR motif have only been reported in haddock [71], Atlantic cod [34] and Siberian sturgeon [31]. However, several mutagenesis studies showed that the modification of ELR motif had no significant impact on the chemotactic activity of CXCL8 in different fish species [21,30,55]. Despite its crucial functional importance in mammals, this observation suggests an ELR motif-independent function for teleost CXCL8s [30,55]. Furthermore, all three aa residues corresponding to the ELR motif were highly variable in teleosts [18]. This extensive variation in fish ELR like motif could be subjected to a functional investigation to understand its biological importance.

In flounder, the chemotactic activity of IL-8 was dependent on a stretch (VSLRSLGV) that precedes the ELR-like (SLH) motif, where the sixth Leu residue was proposed to be critically important for its function [55]. MSA revealed that OfCXCL8-L1 possessed this Leu residue, while OfCXCL8-L3 and few other IL-8s lacked it. Following the ELR motif variant, OfCXCL8s contained a conserved CXC motif, in which two Cys residues were separated by an Arg residue. Altogether, they had four cysteine residues that formed two disulfide bonds required for maintaining the tertiary folding and structural integrity.

Evidence has been clearly established for the existence of three distinct CXCL8 lineages in teleosts [11,14,15,18,23,24,25,70]. A recent study performed detailed evolutionary analyses and suggested that two sub-lineages are present within CXCL8-L1 [70]. Analysis of two cyprinoid genomes, including zebrafish and carp [14,24,72], revealed a cyprinoid-specific CXCL8 lineage 2 (CXCL8-L2), and based on evidence; a gene-specific sub-functionalization was proposed [14]. L1 is composed of teleost-specific IL-8 members, which has been identified from many bony fishes, such as Japanese flounder [12], common carp [13,14,15], rainbow trout [16], and zebra fish [17]. In contrast, L2 members have only been reported from carp and zebrafish and proposed to be a cyprinid-specific lineage [25]. Our study adds a member to the recently identified growing L3 that already had CXCL8 orthologs from large yellow croaker [26] and rainbow trout [11]. Based on evolutionary evidence, a study has demonstrated the correlation between ELR-like motif and CXCL8 lineages [70]. It has been proposed that ancestral ELR-like motif in fish had evolved from the GGR motif of lamprey, and subsequent evolution progressed in two distinct directions to generate a motif with a consensus sequence of (a) EXH/R in L1 (e.g., ELR, DLR) and (b) NXH/R in L3 [70].

A closer look at the BLAST results indicated that the previously submitted sequence (accession number: AB703273; [54]) is not a true CXCL8 ortholog. Our homology and phylogeny analyses revealed that the two *Ofcxcl8*s identified in the present study indeed belong to L1 and L3. While OfCXCL8-L1 demonstrated higher sequence homology with orthologs of L1 compared to L2 and L3, OfCXCL8-L3 showed higher homology with orthologs of L3 than L1 and L2. It was interesting to note that OfCXCL8-L1 shared a relatively higher identity with tetrapod sequences than OfCXCL8-L3 (Table 1). The topology of the phylogenetic tree was consistent with these results (Figure 3), where tetrapod (ELR+) CXCL8s and teleost CXCL8-L1 shared a node, leaving the CXCL8-L3 as the root of the tree. When CXCL8 orthologs of L2 from cyprinids were also included in the evolutionary analyses, they were closely placed with mammalian counterparts within the tetrapod CXCL8 cluster (Figure S3), as reported in previous studies [14,23]. Our results suggest that L3 is closer to the ancestor of CXCL8 (i.e., slowly-evolving), in contrast to the rapidly evolving L1, which is evolutionarily closer to recently evolved tetrapod CXCL8 orthologs, and this is in agreement with the hypothesis proposed by Gangele et al. [70].

The above hypothesis was further validated by the inter-species genomic structural comparison conducted with vertebrate *cxcl8*s of different origins (Figure 4). A previously well-established typical genomic arrangement of *cxcl8* was observed in all the tetrapod- and L1 (including *Ofcxcl8-L1*) of several teleost-species. The exon/intron arrangement was essentially demonstrated to be the same with quadripartite structure, in which four exons are separated by three introns with widely varying sizes. However, members of L3 (including *Ofcxcl8-L3*) had a tri-exonic structure that contained three exons split by two introns. This study, therefore, described a novel member of L3 in teleost *cxcl8*s, which is evolutionarily and genomic structurally distinct from all the previously characterized teleost *cxcl8*s. The *cxcl8-L3* is a newly growing subclass with *cxcl8-like* sequences in teleost fish [26,70], and this study established the general gene structure of teleost *cxcl8-L3* for the first time. Identification of similar orthologs from different teleost species at the genomic level may confirm our findings. Multiple copies of *cxcl8* in teleost might have arisen from whole-genome or chromosome duplication events [18,25,73]. However, detailed studies have to explore whether their functional attributes also vary from each other.

Mechanisms of both basal- and induced-gene expression of *cxcl8* are well understood phenomena in mammalian models. The essence of these findings highlighted that the most important characteristic feature of *cxcl8* is its dynamic expression under different circumstances [74]. However, the factors underlying the molecular transcriptional regulation of *cxcl8* are poorly studied in fish [34,75]. Transcriptional control regions lying upstream of the TIS of *Ofcxcl8*s were obtained by genomic cloning (Figure 5). As a core promoter feature, both *Ofcxcl8*s possessed a TATA box (or a variant), which is located ~25 bp upstream of their TIS, as observed in mammals [76]. Mutagenesis and deletion studies had demonstrated that NF-κB subunits act as the chief regulators of *cxcl8* [76,77]. Synergism and cooperativity between NF-κB and C/EBP have been established as the distinct features in the regulation of *cxcl8* expression [78,79]. Other studies had also suggested that NF-κB and AP-1 cooperatively regulate the induction of *cxcl8* [76,80]. Other than these positive regulators, *cxcl8* gene expression is repressed in resting cells through several negative regulators, including NF-κB-repressing factor (NRF) and octamer-1 (Oct-1) [74]. Screening the 5’-flanking region of *Ofcxcl8*s revealed the presence of two NF-κB elements in *Ofcxcl8-L1* and an NF-κB1 element in *Ofcxcl8-L3* in the vicinity of their TIS. In addition, several C/EBPα, C/EBPβ, Jun:Fos (AP1), and POU2F1 (Oct-1) TFBS were also located in the promoter regions of *Ofcxcl8*s. These findings, together with data obtained in rainbow trout [75,79], provided the fundamental clue regarding the transcriptional regulatory mechanisms of *cxcl8* in teleosts.

We investigated the distribution of *Ofcxcl8* transcripts in different tissues of juvenile rock breams. In mammals, although the primary CXCL8 sources are monocytes and macrophages, virtually all the nucleated cells express CXCL8 [58]. Both *Ofcxcl8* transcripts were widely distributed in several tissues of rock bream at different levels (Figure 6). The predominant level of *Ofcxcl8-L1* transcript was detected in gills, followed by intestine and PBCs. The *cxcl8* of haddock [71] and carp [13] also exhibited their highest mRNA levels in gills. Although the transcripts of *Ofcxcl8-L3* was significantly higher in gills, spleen had the dominant levels of *Ofcxcl8-L3* mRNA, as reported in rainbow trout [16] and sea perch [28]. In contrast, other species demonstrated an entirely different expression pattern; for instance, grass carp and large yellow croaker *cxcl8* orthologs were highly transcribed in the liver [18,22]. Skin, gills, and intestine constitute the first line of protective barrier against pathogens, and abundant *Ofcxcl8* mRNA levels in these tissues may have resulted from resident leukocytes. In accordance with the majority of the previous reports, *Ofcxcl8* homologs were mainly expressed in immune relevant organs under physiological conditions. Moreover, inconsistent tissue profile patterns between studies might be the outcome of various factors, including differences in species, developmental stage, and their physiological state.

Flagellin is a foremost pro-inflammatory determinant and the only known ligand of TLR5. In animal models and cell lines, it has been shown that FLA-mediated TLR5 signaling could activate NF-κB, the chief TF, which governs the expression of a wide array of pro-inflammatory genes, including *cxcl8* [81,82,83,84]. Unfortunately, inflammatory events induced by FLA were not well understood in fish. We investigated the *Ofcxcl8* expression following an in vivo FLA-ST injection (Figure 7). A significantly induced response of *Ofcxcl8-L1* was detected in head kidney, intestine, kidney, gills and liver, whereas it had no significant changes in spleen. On the other hand, transcription of *Ofcxcl8-L3* increased in examined tissues except in liver. Basu et al. examined the expression of mrigal *cxcl8* mRNA expression post-FLA-injection [85]. Our findings are generally in agreement with mrigal *cxcl8* expression profiles. Changes in the *Ofcxcl8* transcription were studied in vitro in PBLs stimulated with Con A, which is an antigenic-stimulant used in studying cellular responses. Among the two homologs, only *Ofcxcl8-L1* demonstrated an inductive transcriptional profile (Figure S2). A rock bream *cxc* was reported to be up-regulated by Con A treatment in leukocytes [54]. We earlier found that *Ofcxcr2*, but not *Ofcxcr1*, was responsive to Con A treatment [10]. Together with previous findings, our data suggest that teleosts could elicit pro-inflammatory responses against FLA and Con A, possibly through their induced cytokines such as *Ofcxcl8*.

As a pro-inflammatory chemokine, CXCL8 exerts its function in leukocytes and endothelial cells via their CXCR1 and CXCR2 receptors to promote immune infiltration and angiogenesis. Earlier reports indicated an induced transcriptional response of the *cxcl8-*L1 in several bony fish species, in response to different PAMP and/or pathogenic bacteria [13,21,22,34]. The parasitic infection has also been reported to cause transcriptional induction of *cxcl8* in teleosts [13,86]. In the current study, *Ofcxcl8-L1* homolog was strongly up-regulated in spleen upon LPS- and bacterial-challenges (Figure 8A–C). Based on magnitude of fold-change, Gram-negative bacterium (i.e., *E. tarda*) and LPS, which is a structural constituent of Gram-negative strains, had a greater impact on *Ofcxcl8-L1* expression compared with Gram-positive bacterium (i.e., *S. iniae*) suggesting that Gram-negative bacteria appear to be the potent stimulant of *Ofcxcl8-L1* transcription. This fact corroborates with the previous findings reported in different fish-pathogen models, including mrigal against *Aeromonas hydrophila*/*E. tarda* [85], grass carp against *A. hydrophila* [18], turbot against *E. tarda* [21], Siberian sturgeon against *A. hydrophila* [31] and large yellow croaker against *Vibrio parahaemolyticus* [22] infections.

In terms of the magnitude of *Ofcxcl8-L1*-induction, LPS and bacteria elicited a strong effect compared to that of poly(I:C) and RBIV, indicating that the bacterial induction of *Ofcxcl8-L1* was stronger than that of viral induction (Figure 8). While the cod *il-8* maintained a stable basal expression in head kidney cells upon in vitro PAMP- stimulation and IPNV virus treatment, in vivo injection of *V. anguillarum* or poly(I:C) resulted in a strong induction in *il-8* transcript levels [34]. An inactivated trivalent bacterial vaccine induced *Lyccxcl8-L3* mRNA expression in large yellow croaker spleen [26]. In contrast, *Ofcxcl8-L3* was transcriptionally non-responsive against the challenges we performed, except for poly(I:C), where it demonstrated a statistically significant but weak response (Figure 8D). Relative fold-change in *Ofcxcl8-L1* transcripts against PAMP or pathogen challenges was higher in general compared to that of *Ofcxcl8-L3*. These findings imply that, among the two *Ofcxcl8* homologs, only *Ofcxcl8-L1* could be strongly inducible, and hence, it may exhibit potential pro-inflammatory response, particularly during the bacterial infections.

Mammalian ELR+ and ELR- chemokines play distinct roles by targeting different cell types. Human CXCL8, an ELR+ chemokine, induces chemotaxis, primarily in neutrophils, but also in other granulocytes and T lymphocytes [7]. The chemotactic activity has been demonstrated using rCXCL8 in different fish species (e.g., rainbow trout [32,33], carp [14,15], half-smooth tongue sole [20], turbot [21], large yellow croaker [23,26], black seabream [29], grass carp [18], ayu [19], Siberian sturgeon [31], and zebrafish [24]) using heterogeneous (i.e., leukocytes) and/or different homogeneous cell types, such as neutrophils, macrophages, and lymphocytes from head kidney and PBLs. In order to investigate the chemotactic and proliferative properties, the mature OfCXCL8s were recombinantly expressed in bacteria in the form of MBP-tagged proteins and purified. We used an enriched kidney leukocyte model to characterize the biological activities of rOfCXCL8s (Figure 9). In the present study, both rOfCXCL8-L1 and rOfCXCL8-L3 induced leukocyte chemotaxis and proliferation. However, rOfCXCL8-L1 exhibited robust activities compared to that of rOfCXCL8-L3, where the activities of rOfCXCL8-L3 were not significantly different from rMBP control. We speculate that multiple factors might have contributed to these differences in their biological activities: (1) EMH motif in rOfCXCL8-L1 is biochemically similar to ELR motif, whereas NSH motif in rOfCXCL8-L3 is relatively different; (2) Leu residue preceding the ELR motif, which is considered essential for the function of CXCL8 [55], is only conserved in rOfCXCL8-L1. However, it should be noted that we employed fusion proteins in this study, and the impact of the presence of a fusion partner (MBP-tag) on biological activities of recombinant CXCL8 is not known. Regardless of the absence of ELR motif, CXCL8 orthologs from rock bream and several other teleosts have demonstrated chemotactic [14,18,20,21,23,29] and proliferative [20,21,31] functions, indicating that the ELR motif may not be a structural prerequisite for the biological properties of CXCL8 in fish. In turbot CXCL8, replacing the EMH motif with AAA motif through mutagenesis led to the severe impairment in chemotactic capacity [21], suggesting that EMH motif may also be important for the function of rOfCXCL8-L1. Unlike mammalian CXCL8s with chemotactic function inducing migration of specific cell types (e.g., neutrophils), fish CXCL8s are capable of inducing migration of different target cells. Although we have already identified the receptors for CXCL8 in rock bream [10], their roles in transducing signals from OfCXCL8 homologs require further research.

## 5. Conclusions

In summary, we have identified, cloned, and characterized two homologs of *cxcl8* belonging to lineage 1 and a recently proposed novel lineage 3 of teleost *il-8*s from *O. fasciatus*, *Ofcxcl8-L1*, and *Ofcxcl8-L3*. For the first time, we documented the putative tripartite exonic organization of the member of lineage 3 (i.e., *Ofcxcl8-L3*), which varied from the highly-conserved tetrapartite exonic structure of lineage 1 members. Two *Ofcxcl8* homologs also demonstrated distinct characteristics in terms of their translated aa sequences, evolutionary aspects, expressional patterns in normal and immune-challenged animals, and biological functions assayed for chemotaxis and proliferation using rOfCXCL8s. Differential involvement of these homologs in pro-inflammatory response was evidenced based on their transcriptional expression and functional properties. *Ofcxcl8-L1* was more inducible, and rOfCXCL8-L1 exhibited relatively high chemotactic and proliferative capacity compared to *Ofcxcl8-L3* and its recombinant protein, respectively. Collectively, our lines of evidence support the roles of two *Ofcxcl8* homologs in the inflammatory responses, and the present study contributes to broadening our current understanding of piscine chemokines.

## Figures and Tables

**Table 1:** Homology indices of rock bream CXCL8s (OfCXCL8-L1 and OfCXCL8-L3) with known selected orthologs of other vertebrates.

Taxonomy	Species	Accession No	Protein Name	OfCXCL8-L1		Species	Accession No	Protein Name	OfCXCL8-L3	
				I%	S%				I%	S%
Fish	*Oplegnathus fasciatus*	AGR27882.1	Interleukin-8 (OfCXCL8-L1)	100.0	100.0	*Oplegnathus fasciatus*	AGR27882.1	Interleukin-8 (OfCXCL8-L1)	30.60	53.30
Fish	*Oplegnathus fasciatus*	ADK35757.1	Interleukin-8	98.0	99.0	*Oplegnathus fasciatus*	ADK35757.1	Interleukin-8	29.70	53.30
Fish	*Oplegnathus fasciatus*	AGR27883.1	Interleukin-8 like (OfCXCL8-L3)	30.6	53.3	*Oplegnathus fasciatus*	AGR27883.1	Interleukin-8 like (OfCXCL8-L3)	100.00	100.00
Fish	*Siniperca chuatsi*	AFK65606.1	Interleukin-8	88.9	94.9	*Lateolabrax japonicus*	QFG40053.1	Interleukin-8	91.40	96.20
Fish	*Dicentrarchus labrax*	CAM32186.1	Interleukin-8	87.9	93.9	*Sparus aurata*	XP_030285781.1	Permeability factor 2	89.50	96.20
Fish	*Latris lineata*	ACQ99511.1	Interleukin-8	86.9	94.9	*Lutjanus peru*	ASK51661.1	Interleukin-8	89.50	95.20
Fish	*Pagrus major*	ADK35756.1	Interleukin-8	85.0	92.0	*Dicentrarchus labrax*	AKC57337.1	Interleukin-8	87.60	94.30
Fish	*Anoplopoma fimbria*	ACQ57874.1	Interleukin-8	79.8	91.9	*Salarias fasciatus*	XP_029943750.1	Interleukin-8	81.90	89.50
Fish	*Cyprinus carpio*	ABE47600.1	Interleukin-8	60.6	77.6	*Anoplopoma fimbria*	ACQ58275.1	Interleukin-8	78.10	83.80
Fish	*Danio rerio*	XP_001342606.2	Permeability factor 2 IF X1	58.6	76.5	*Danio rerio*	XP_001342606.2	Permeability factor 2 IF X1	29.70	55.20
Fish	*Oncorhynchus mykiss*	AAO25640.1	Interleukin-8	57.1	76.5	*Tetraodon nigroviridis*	CAF90539.1	Unnamed protein *	68.60	81.90
Amphibia	*Xenopus tropicalis*	XP_002942577.1	Interleukin-8	39.3	60.0	*Xenopus tropicalis*	XP_002942577.1	Interleukin-8	31.20	52.40
Amphibia	*Xenopus laevis*	AEB96252.1	Interleukin-8	37.1	63.1	*Xenopus laevis*	AEB96252.1	Interleukin-8	33.90	55.20
Reptilia	*Anolis carolinensis*	XP_003230087.1	Interleukin-8	41.2	65.0	*Anolis carolinensis*	XP_003230087.1	Interleukin-8	33.90	50.50
Aves	*Gallus gallus*	NP_990349.1	Interleukin-8	41.5	63.5	*Gallus gallus*	NP_990349.1	Interleukin-8	30.30	53.30
Mammalia	*Sus scrofa*	NP_999032.1	Interleukin-8	34.3	56.3	*Sus scrofa*	NP_999032.1	Interleukin-8	27.50	49.50
Mammalia	*Homo sapiens*	NP_000575.1	Interleukin-8 IF 1	34.7	57.6	*Homo sapiens*	NP_000575.1	Interleukin-8 IF 1	25.20	56.20
Mammalia	*Oryctolagus cuniculus*	NP_001075762.1	Interleukin-8	33.0	56.4	*Oryctolagus cuniculus*	NP_001075762.1	Interleukin-8	27.50	52.40

Matrix was generated by the MatGAT program using BLOSUM62 scoring matrix, maintaining the first gap penalty and extending gap penalty levels at 12 and 1, respectively. IF, isoform. * Blast hit showed that this is an interleukin-8 homolog and phylogeny analyses showed it belongs to lineage 3 (L3).

**Table 2:** Inter-lineage genome organization and comparison of exon-intron structure for classic *il-8* and novel *il-8-like* genes among vertebrate classes.

Gene Symbol ^1^	Accession Number/Reference	Organism	Exon 1 ^3^		Intron 1	Exon 2	Intron 2	Exon 3 ^4^	Intron 3	Exon 4 ^4^	Group	
			5′ UTR	CDS^P^		CDS		CDS		CDS^X,Q^	3′ UTR	
*cxcl8*	ENST00000307407 ^2^	*Homo sapiens*	153	64	819	136	271	84	416	16	1252	Mammals
*cxcl8*	ENSBTAT00000026275 ^2^	*Bos taurus*	70	64	1573	136	273	84	439	22	1105	Mammals
*cxcl8*	ENSSSCT00000009807 ^2^	*Sus scrofa*	83	64	1018	136	288	84	427	28	1093	Mammals
*cxcl8*	ENSOCUT00000030077 ^2^	*Oryctolagus cuniculus*	265	64	516	136	269	84	448	22	1148	Mammals
*il-8*	ENSGALT00000042745 ^2^	*Gallus gallus*	-	61	774	136	642	84	568	31	2661	Birds
*il-8 like*	XM_005498533	*Columba livia*	63	61	773	136	700	84	1249	31	169	Birds
*il-8like*	ENSACAT00000011352 ^2^	*Anolis carolinensis*	-	64	993	136	265	80	339	32	-	Reptiles
*il-8*	XM_004911120	*Xenopus tropicalis*	81	64	263	124	676	88	1490	12	294	Amphibians
*Ofcxcl8-L1*	This study	*Oplegnathus fasciatus*	200	67	194	133	146	87	142	10	358	Fish
*il-8*	Laing et al. (2002)	*Oncorhynchus mykiss*	-	64	341	133	247	87	292	10	-	Fish
*il-8*	ENSTRUT00000016751 ^2^/NC_042301	*Takifugu rubripes*	137	64	107	133	93	87	106	12	365	Fish
*il-8*	Wang et al. (2013)	*Ctenopharyngodon idella*	-	64	143	133	123	87	125	13	-	Fish
*il-8 precursor*	EU007442/ Seppola et al. (2008)	*Gadus morhua*	122	73	109	133	151	87	202	13	169	Fish
*il-8*	JQ407041/ Li et al. (2013)	*Larimichthys crocea*	-	52	168	133	149	87	682	13	-	Fish
*il-8*	KP202400/ Mu et al. (2015)	*Larimichthys crocea*		64	137	133	129	87	124	16	-	Fish
*cxca*	AJ421443/ Huising et al. (2003)	*Cyprinus carpio*	81	58	117	133	126	88	148	19	247	Fish
*cxcl8b.1*	XM_003198892/NM_001327985	*Danio rerio*	74	70	79	130	2660	69	2476	88	303	Fish
*il-8*	Chen et al. (2005)	*Ictalurus punctatus*	-	67	87	136	153	69	164	73	-	Fish
*il-8 like*	ENSTNIT00000005982^2^	*Tetraodon nigroviridis*	-	52	427	136	140	130	-	-	-	Fish
*il-8 like x2*	NW_004531887/XM_004570701	*Maylandia zebra*	46	52	1090	136	1252	121	-	-	413	Fish
*Ofcxcl8-L3*	This study	*Oplegnathus fasciatus*	59	52	2202	136	2209	130	-	-	625	Fish

^1^ As provided in the respective database resource; ^2^ Ensembl accession number; ^3^ the first (Exon 1) and ^4^ last (Exon 3 or 4) have been divided into two parts (5′- or 3′-UTRs and CDS); exons and parts of exons composing the CDS of *cxcl8* are gray shaded; the exons in CDS, those deviating from the generalized structure (Figure 4), are underlined; X, non-conserved exon (a segment in exon 4) in tetrapod *cxcl8*, P, Q, non-conserved exons (segments in exon 1 and 4, respectively) in classic *cxcl8* of teleosts.

**Table 3:** Concise comparison of the properties of rock bream *cxcl8*s, *Ofcxcl8-L1*, and *Ofcxcl8-L3*, at different molecular levels.

	Characteristics	*Ofcxcl8-L1*	*Ofcxcl8-L3*
Complimentary DNA	GenBank accession No.	KC522966	KC522965
	Length of cDNA	855	1002
	5′ UTR	200	59
	CDS (bp)	297	318
	3′ UTR	358	625
	mRNA instability motif	3	2
	Polyadenylation signal	^831^AATAAA^836^	^986^AATAAA^991^
Genome	Length of gDNA (bp)	1337	5413
	Number of exons	4	3
	Number of introns	3	2
Protein	Peptide (aa)	98	105
	Molecular mass (Da)	10,833	11,571.6
	Theoretical *p*I	8.78	8.95
	Signal peptide	1–23	1–18
	SCY domain	32-93	23-88
	ELR-like motif	^32^EMH^34^	^27^NSH^29^
	CXC motif	^35^CRC^37^	^30^CRC^32^
	Invariant cysteines	35, 37, 61, 78	30, 32, 57, 73
	Highest homology	Mandarin fish IL-8	Japanese sea bass IL-8
5′-flanking region	Transcription factor binding sites	NF-κB/ NF-κB1, C/EBPα, C/EBPβ (NF-IL6), Jun:Fos (AP1) and POU2F1 (Oct-1)	
Transcripts	Dominant mRNA expression (qPCR)	gills, intestine, and PBCs	spleen and gills
Functional characteristics	Chemotaxis index (100 ng/µL) Cell proliferation (OD_450_)	~130 (significant) Significant at 10 and 100 ng/µL	~19 (not significant) Not significant

## Supplementary Materials

The following are available online at https://www.mdpi.com/2218-273X/10/10/1382/s1, Figure S1: Nucleotide and amino acid sequences of rock bream interleukin-8 sequences, (**A**) OfCXCL8-L1 and (**B**) OfCXCL8-L3; S2: The temporal mRNA expression of rock bream *cxcl8-L1* (*Ofcxcl8-L1*) and *cxcl8-L3* (*Ofcxcl8-L3*) in PBLs, following Con A treatment detected by SYBR green qPCR; S3: Sodium dodecyl sulfate-polyacrylamide gel electrophoresis (SDS–PAGE) analysis of recombinant rock bream OfCXCL8 (rOfCXCL8) fusion proteins, (**A**) rOfCXCL8-L1 and (**B**) rOfCXCL8-L3, sampled at different steps of amylose resin affinity chromatography; S4: Molecular phylogenetic tree of selected CXCL8 (IL-8) homologs from vertebrates, Table S1: Description of primers used in this study; S2: Details of immune challenge experiments conducted to examine the transcriptional expression of *Ofcxcl8* homologs in the current study; S3: Identity and similarity matrix showing the identity/similarity percentages between OfCXCL8 homologs with two cyprinid IL8 lineages at amino acid level.
