# Supplementary material for: Comparative Characterization of Two cxcl8 Homologs in Oplegnathus fasciatus: Genomic, Transcriptional and Functional Analyses
Source: Biomolecules. 2020 Sep 28;10(10):1382. doi: 10.3390/biom10101382 (PMC7601086; doi:10.3390/biom10101382)
Supplement: Supplementary file 1 [file biomolecules-10-01382-s001.pdf]

biomolecules

MDPI

Article

Comparative Characterization of Two *cxcl8* Homologs in *Oplegnathus fasciatus*: Genomic, Transcriptional and Functional Analyses

Navaneethaiyer Umasuthan [1,2,*], SDNK Bathige [1,3], William Shanthakumar Thulasitha[1,4], Minyoung Oh[1,5] and Jehee Lee [1,6,*]

1 Department of Marine Life Sciences & Fish Vaccine Research Center, Jeju National University, Jeju Self-Governing Province 63243, Republic of Korea
2 Department of Ocean Sciences, Memorial University of Newfoundland, St. John's, NL A1C 5S7, Canada
3 Sri Lanka Institute of Nanotechnology (SLINTEC), Nanotechnology and Science Park, Mahenwatta, Pitipana, Homagama, Sri Lanka
4 Department of Zoology, Faculty of Science, University of Jaffna, Jaffna 40000, Sri Lanka
5 Department of Biochemistry and Molecular Biology, Dalhousie University, Halifax, NS, Canada
6 Marine Science Institute, Jeju National University, Jeju Self-Governing Province, 63333, Republic of Korea
* Correspondence: navaumasuthan@gmail.com (NU); jehee@jejunu.ac.kr (JL)

Supplementary Figures

(A)

```
  1                                                 GATTCAAACAGCAGAGCAAG
 21   AAAGCAGCTGAACCAAGAAAAGGAAAAGTAGCGGAGAGAGGGAACTGAGTGAAAGAAGAG
 81   AGAAGAAAAAAAAAAGACCATCTTTTTACTTTCACACAACGCTTCAGGCTTCATCAGGCAG
141   CTTTCTGAAGGACATTCCTATCCTTTAGTGTAAACTGTTGCAGAATTTCTGAAAGACAAA
201   atgatgagcagcaaagttatcgtcatctctgtcgtggtgctcctggccttcctggccatc
      M  M  S  S  K  V  I  V  I  S  V  V  V  L  L  A  F  L  A  I    20
261   agtgaagggatgagtctgagaagtctgggaatggagatgcactgccgctgcatccagaca
      S  E  G  M  S  L  R  S  L  G  M  E  M  H  C  R  C  I  Q  T    40
321   gagagcaaacccatcggccgccacatcgggaaggtggagctgattcctgccaactcccac
      E  S  K  P  I  G  R  H  I  G  K  V  E  L  I  P  A  N  S  H    60
381   tgcgaggagactgagatcattgccactctgaaaaagacaggccaagaggtttgcctggac
      C  E  E  T  E  I  I  A  T  L  K  K  T  G  Q  E  V  C  L  D    80
441   cccgaggctccctgggtgaagaaagtgattaaggcgatcatgaacaaaagtcgctgaACA
      P  E  A  P  W  V  K  K  V  I  K  A  I  M  N  K  S  R  *      98

501   GACCGGGAGAGATGTGTTTCATGAGCCTTAGCTGCTCATAAACAGATGAAAAGTACCAAA
561   AAGTATTTGTTTGGTGATGATAACGTCATCACATGTCATACCAGAGGACCACTTAATGTT
621   CGGATCAACTGTTGAAAATAACAACAGAATGTATCCACTGTATGTTTGTATCAAACATGT
681   TATTTATTACCAAGTCTGTGCTTGTTTGTATGTCACTTATGTGCATTACTATATATATAA
741   CTTATGTGTTTAACATATTTATTGATGTATTTATGAGATGTAAATGTATCCAGATTCAGT
801   TGTTTATCTCTGCTGACAATCTACTGATCAAATAAAAAAAGTTGATGTGAAATTG
```

(B)

```
  1    CAACAGCTTCATTATATCTGTCTGAGTTAAAGAACTGAAGAAGCAGCTGTGAAAACAAG
 60   atgaagctctgcgtcctcctgatttctgggaccttgcttgtcctcattaacggtatgccg
      M  K  L  C  V  L  L  I  S  G  T  L  L  V  L  I  N  G  M  P    20
120   ccaatcagcagggactacaattcacactgccggtgcctgcaggtggagtcgaggatcatc
      P  I  S  R  D  Y  N  S  H  C  R  C  L  Q  V  E  S  R  I  I    40
180   cctccagacaacctgaggagtatcaagctcgtccctgaagggccccactgcccagaaaca
      P  P  D  N  L  R  S  I  K  L  V  P  E  G  P  H  C  P  E  T    60
240   gaagtcatagctggactggccagcggggagaaggtttgcttgaaccctcggtcctcctgg
      E  V  I  A  G  L  A  S  G  E  K  V  C  L  N  P  R  S  S  W    80
300   gtgaagaagctgatccactttgttcttgagaaacagctacatcagcagggtggagcacct
      V  K  K  L  I  H  F  V  L  E  K  Q  L  H  Q  Q  G  G  A  P   100
360   cccaagaatcaagcataaTATCATGAAGCAAATCAAAAACCTTTTGCAATTAAAGCTGTT
      P  K  N  Q  A  *                                             105
420   GAAAATGACTCCTTTCTGCAACTATGAGCCATGCTGTTTTACTTTAATGGATAAATATTT
480   AACCAAGTTTGTGTAACTTAAATAACTTCTTTTTCATTTTTTTTCTGGTTTCTGTTGTTAG
540   TGTTATTTCAGCCTTTTTGTGTTTTAATTTCAGAAACTGTTTTTTATCAGATATTTTATA
600   CACATTTTCTAAATGACGTTGATTCTGGATTTATATTGTTTAAGTGTAAGAAAGAGCACA
660   CAAAAGCAGATGTGTGTGGCTGTGCTAGGAAGTAAATGGACAGTAAGCAATCAGCTGTAA
720   TGTAAAAGTGTTGGAAGGCTTTGTGTGCATTAAAAAGTCATGTGTGACTTGAAATGTAAA
780   TCAATTCTCTGTGTAGAAAGTTTGATTGTATTACCTTTTGAAAGCAGATACTGCCATGTA
840   GCACGTAACATGTAATATTTCTGTGTGATACTCCTTTTGATAAAGCTTCTCCAACAATAG
900   CAAGAAACAATCAGTTCCTTTGTTCCCTAGTCCCTAACTAAGGACTTTTATAAGAAAGAA
960   CTGAAAGATGTAATGTGAGGCTGAAAAATAAAAACAAATATAT
```

Figure S1. Nucleotide (black) and amino acid (blue) sequences of rock bream interleukin-8 sequences, (**A**) OfCXCL8-L1 and (**B**) OfCXCL8-L3. In DNA sequence: UTRs are in upper case, while CDS is in lowercase. The RNA instability motifs are underlined and italicized. The canonical polyadenylation signal site is bold italicized. In amino acid sequences: Signal peptide is underlined by dot line, SCY domain (CXC chemokine module; yellow), ELR motif variants (NSH or EMH; red), and CXC signature (CXC; purple) are shown. Four cysteine residues are marked.

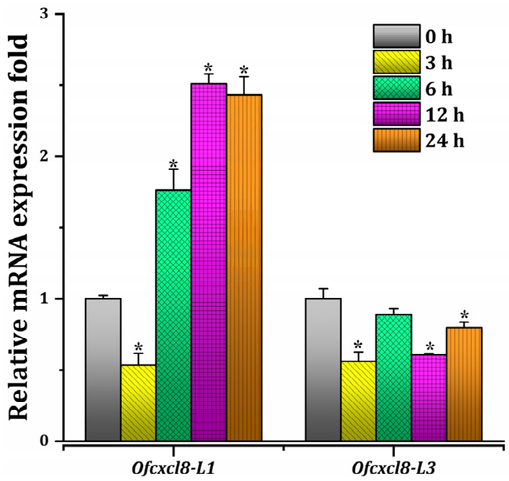

Figure S2. The temporal mRNA expression of rock bream *cxcl8-L1* (*Ofcxcl8-L1*) and *cxcl8-L3* (*Ofcxcl8-L3*) in PBLs, following Con A treatment detected by SYBR green qPCR. The vertical bars represent S.D. (n=3). For details of the captions, please refer to **Figure 7**.

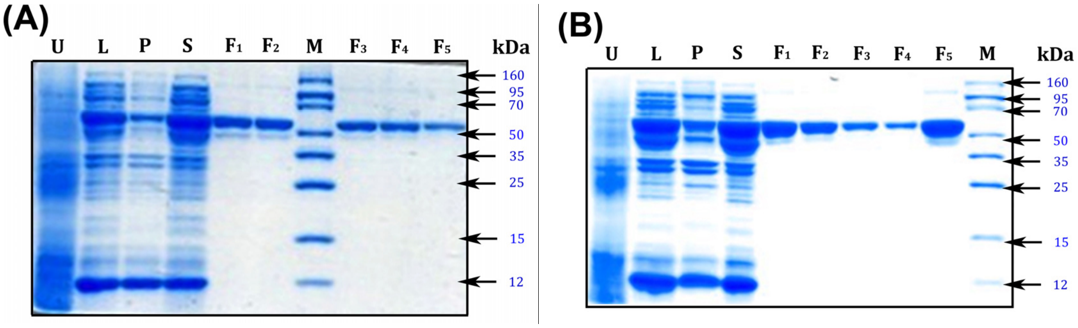

Figure S3. Sodium dodecyl sulfate-polyacrylamide gel electrophoresis (SDS–PAGE) analysis of recombinant rock bream OfCXCL8 (rOfCXCL8) fusion proteins, (**A**) rOfCXCL8-L1 and (**B**) rOfCXCL8-L3, sampled at different steps of amylose resin affinity chromatography. Exponentially grown *E. coli* BL21 (DE3) cells harboring recombinant Ofcxcl8/ pMAL-c2X vectors were exogenously induced with 0.3 mM IPTG at 17 °C for 12 h. Recombinant OfCXCL8 proteins were extracted using pMAL™ protein fusion and purification system protocol (New England Biolabs, Inc). Lanes: (U), total proteins in cellular extract prior to IPTG induction; (L), total proteins in cellular lysate after IPTG induction; relative distribution of rOfCXCL8 proteins in (P) insoluble and (S) soluble fractions after cell lysis by sonication; (F_1–F_5) purified rOfCXCL8 fusion proteins in different fractions eluted; (M) molecular standards (Enzynomics).

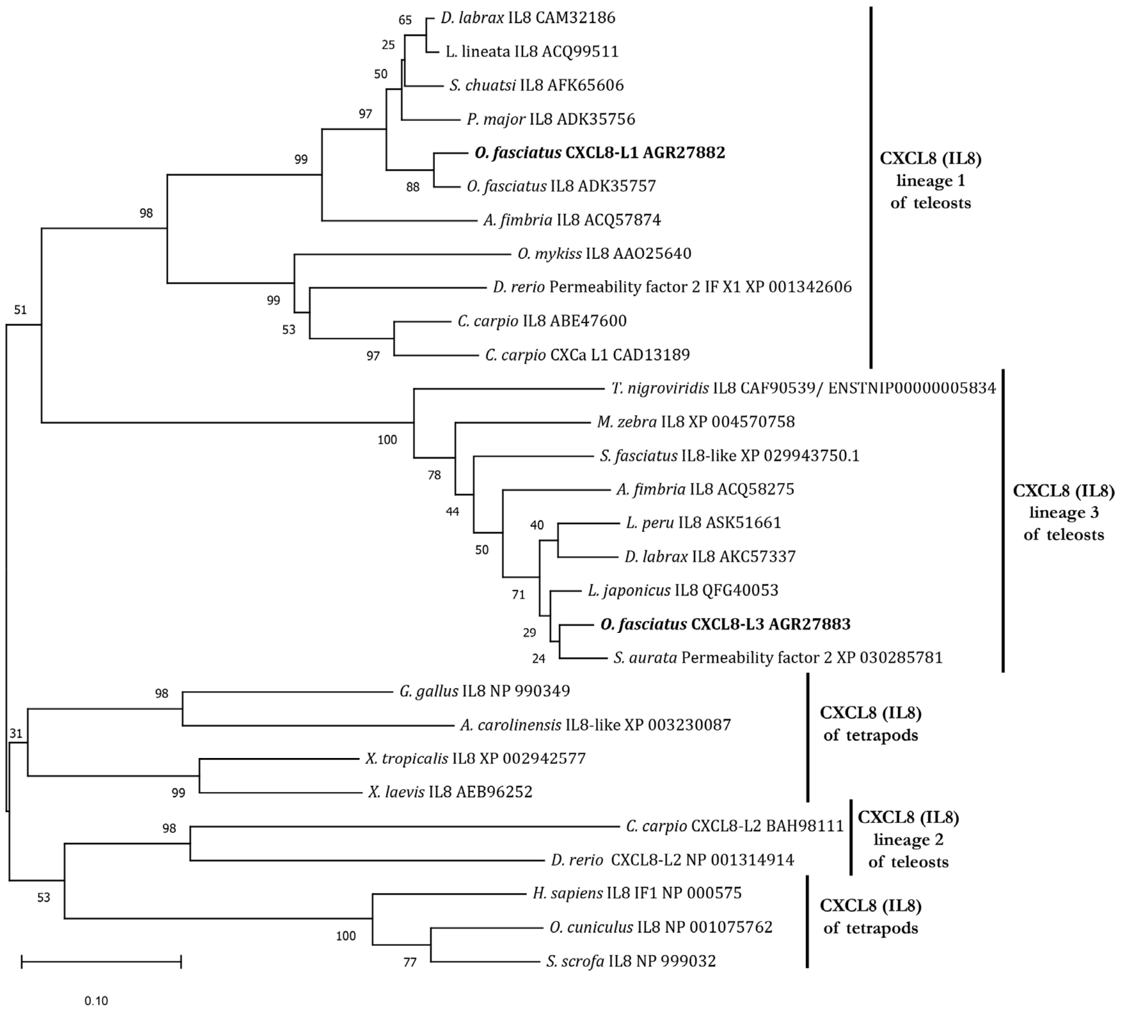

Figure S4. Molecular phylogenetic tree of selected CXCL8 (IL-8) homologs from vertebrates. The evolutionary history was inferred by the Neighbor-Joining method, and the evolutionary distances were computed with the p-distance method using MEGA X. Major clusters are indicated with vertical bars. The values at the forks indicate the percentage of trees in which the grouping occurred after bootstrap 5000 replicates. The tree was drawn to scale. The GenBank accession numbers are given next to each species. For tree depicted in Figure 3, lineage 2 CXCL8 homologs from teleosts have been included to generate this tree.

biomolecules

MDPI

Supplementary Tables

Table S1. Description of primers used in this study.

Identifier	Target	Sequence (5′ to 3′)	Purpose	Amplicon (bp)	Accession No
F1	*Ofcxcl8-L1*	GAGGGAACTGAGTGAAAGAAG	CDS cloning for sequence confirmation	561	KC522966
R1	*Ofcxcl8-L1*	CATTAAGTGGTCCTCTGGTATG	CDS cloning for sequence confirmation		
F2	*Ofcxcl8-L3*	CTGAAGAAGCAGCTGTGAA	CDS cloning for sequence confirmation	907	KC522965
R2	*Ofcxcl8-L3*	CTTAGTTAGGGACTAGGGAACA	CDS cloning for sequence confirmation		
F3	*Ofcxcl8-L1*	CATCCAGACAGAGAGCAAACCCAT	BAC library screening and qPCR assay	126	KC522966
R3	*Ofcxcl8-L1*	AGGCAAACCTCTTGGCCTGTCTTT	BAC library screening and qPCR assay		
F4	*Ofcxcl8-L3*	AGAAGGTTTGCTTGAACCCTCGGT	BAC library screening and qPCR assay	86	KC522965
R4	*Ofcxcl8-L3*	TCCACCCTGCTGATGTAGCTGTTT	BAC library screening and qPCR assay		
F5	*Ofcxcl8-L1*	GAGAGAgaattcATGAGTCTGAGAAGTCTGGGAATGGAGA	CDS cloning for protein expression - EcoRI	228	KC522966
R5	*Ofcxcl8-L1*	GAGAGAaagcttTCAGCGACTTTTGTTCATGATCGCCTTA	CDS cloning for protein expression - HindIII		
F6	*Ofcxcl8-L3*	GAGAGAgaattcATGCCGCCAATCAGCAGGG	CDS cloning for protein expression - EcoRI	264	KC522965
R6	*Ofcxcl8-L3*	GAGAGAaagcttTTATGCTTGATTCTTGGGAGGTGCTCC	CDS cloning for protein expression - HindIII		
F7	*β-actin*	TCATCACCATCGGCAATGAGAGGT	qPCR assay (internal reference)	108	FJ975145
R7	*β-actin*	TGATGCTGTTGTAGGTGGTCTCGT	qPCR assay (internal reference)		

Restriction enzyme target sites in primer sequences are in lowercase and corresponding enzymes are indicated under 'Purpose'.

Table S2. Details of immune challenge experiments conducted to examine the transcriptional expression of *Ofcxcl8* homologs in the current study.

Group No.[1]	PAMP/ pathogen	Source[2]	Dose per fish	Bodyweight range (g)	Stock concentration[3]	n[4]
1	Un-challenged	-	-	50 ± 4 (and 96 ± 8)	-	3 (FLA-ST, 4)
2	PBS (control)	-	-	50 ± 4 (and 96 ± 8)	-	3 (FLA-ST, 4)
3	Flagellin (FLA-ST)	*S. typhimurium* (InvivoGen)	2.4 µg	96 ± 8	2.4×10^{-2} mg/mL	4
4	LPS	*E. coli* 055:B5, Sigma	125 µg	50 ± 4	1.25 mg/mL	3
5	Poly(I:C)	Sigma	150 µg	50 ± 4	1.5 mg/mL	3
6	RBIV	Infected kidney	10^2 $TCID_{50}$	50 ± 4		3
7	*Edwardsiella tarda*	CNU	5×10^5 CFU	50 ± 4	5×10^3 CFU/µL	3
8	*Streptococcus iniae*	CNU	1×10^7 CFU	50 ± 4	1×10^5 CFU/µL	3

[1] Challenges 3 and 4–8 were conducted independently in two different periods, and the corresponding control experiments (1 and 2) were conducted in both contexts. [2] Obtained from the Department of Aqualife Medicine at Chonnam National University (CNU). [3] All the stimulants were resuspended in 1× PBS, and 100 µL of each stimulant was administered. Route of administration was intraperitoneal (i.p.) for all the challenge groups, except for challenge group 6, which was intramuscular (i.m.). [4] From all the experimental groups, three biological replicates (n = 3) were sampled, except for FLA-ST challenge and its corresponding control (PBS) challenge (n=4). PAMP, pathogen-associated molecular pattern; n, number of biological replicates.

Table S3. Identity and similarity matrix showing the identity/similarity percentages between OfCXCL8 homologs with two cyprinid IL8 lineages at amino acid level

No	Protein molecule	1	2	3	4	5	6	Accession No	Species	Lineage	Reference
1	OfCXCL8-L1		30.6	56.4	27.9	58.6	30.1	AGR27882.1	Rock bream	Putative L1	Current study
2	OfCXCL8-L3	53.3		30.6	29.7	29.7	29.8	AGR27883.1	Rock bream	Putative L3	Current study
3	CXCa_L1	72.4	52.4		24.8	74	27.6	CAD13189	Common carp	L1	Van Der Aa et al. (2010)
4	CXCL8_L2	49.5	49.5	42.4		27.5	45.8	BAH98111	Common carp	L2	Van Der Aa et al. (2010)
5	CXCL8_L1	76.5	55.2	83.7	48.5		22.8	XP_001342606	Zebrafish	L1	Van Der Aa et al. (2010)
6	CXCL8_L2	44.9	42.4	42.4	56.8	41.5		NP_001314914	Zebrafish	L2	Van Der Aa et al. (2010)

Matrix was generated by the MatGAT program using the BLOSUM62 scoring matrix, maintaining the first gap penalty and extending gap penalty levels at 12 and 1, respectively. Identity and similarity (%) are shaded with dark gray and light gray, respectively. Please note that L2 is exclusive to cyprinids, and to date, it has only been reported in common carp and zebrafish [1-3].

1. van der Aa, L.M.; Chadzinska, M.; Tijhaar, E.; Boudinot, P.; Verburg-van Kemenade, B.M.L. CXCL8 chemokines in teleost fish: two lineages with distinct expression profiles during early phases of inflammation. *PloS one* **2010**, *5*, e12384.
2. Gangele, K.; Jamsandekar, M.; Mishra, A.; Poluri, K.M. Unraveling the evolutionary origin of ELR motif using fish CXC chemokine CXCL8. *Fish & shellfish immunology* **2019**, *93*, 17-27.
3. Chen, J.; Xu, Q.; Wang, T.; Collet, B.; Corripio-Miyar, Y.; Bird, S.; Xie, P.; Nie, P.; Secombes, C.J.; Zou, J. Phylogenetic analysis of vertebrate CXC chemokines reveals novel lineage specific groups in teleost fish. *Developmental & Comparative Immunology* **2013**, *41*, 137-152.
